# Systems-Level Analysis of Oxygen Exposure in *Zymomonas mobilis*: Implications for Isoprenoid Production

**DOI:** 10.1128/mSystems.00284-18

**Published:** 2019-02-12

**Authors:** Julia I. Martien, Alexander S. Hebert, David M. Stevenson, Matthew R. Regner, Daven B. Khana, Joshua J. Coon, Daniel Amador-Noguez

**Affiliations:** a DOE Great Lakes Bioenergy Research Center, University of Wisconsin—Madison, Madison, Wisconsin, USA; b Department of Bacteriology, University of Wisconsin—Madison, Madison, Wisconsin, USA; c Genome Center of Wisconsin, Madison, Wisconsin, USA; d Department of Biochemistry, University of Wisconsin—Madison, Madison, Wisconsin, USA; e Department of Biomolecular Chemistry, University of Wisconsin—Madison, Madison, Wisconsin, USA; f Department of Chemistry, University of Wisconsin—Madison, Madison, Wisconsin, USA; g Morgridge Institute for Research, Madison, Wisconsin, USA; University of Queensland

**Keywords:** MEP pathway, *Zymomonas mobilis*, biofuels and bioproducts, gluconate, iron-sulfur clusters, isoprenoids, metabolomics, oxygen exposure, proteomics, transcriptomics

## Abstract

Zymomonas mobilis is an aerotolerant anaerobe and prolific ethanologen with attractive characteristics for industrial bioproduct generation. However, there is currently insufficient knowledge of the impact that environmental factors have on flux through industrially relevant biosynthetic pathways. Here, we examined the effect of oxygen exposure on metabolism and gene expression in Z. mobilis by combining targeted metabolomics, mRNA sequencing, and shotgun proteomics. We found that exposure to oxygen profoundly influenced metabolism, inducing both transient metabolic bottlenecks and long-term metabolic remodeling. In particular, oxygen induced a severe but temporary metabolic bottleneck in the methyl erythritol 4-phosphate pathway for isoprenoid biosynthesis caused by oxidative damage to the iron-sulfur cofactors of the final two enzymes in the pathway. This bottleneck was resolved with minimal changes in expression of isoprenoid biosynthetic enzymes. Instead, it was associated with pronounced upregulation of enzymes related to iron-sulfur cluster maintenance and biogenesis (i.e., flavodoxin reductase and the *suf* operon). We also detected major changes in glucose utilization in the presence of oxygen. Specifically, we observed increased gluconate production following exposure to oxygen, accounting for 18% of glucose uptake. Our results suggest that under aerobic conditions, electrons derived from the oxidation of glucose to gluconate are diverted to the electron transport chain, where they can minimize oxidative damage by reducing reactive oxygen species such as H_2_O_2_. This model is supported by the simultaneous upregulation of three membrane-bound dehydrogenases, cytochrome *c* peroxidase, and a cytochrome *bd* oxidase following exposure to oxygen.

**IMPORTANCE** Microbially generated biofuels and bioproducts have the potential to provide a more environmentally sustainable alternative to fossil-fuel-derived products. In particular, isoprenoids, a diverse class of natural products, are chemically suitable for use as high-grade transport fuels and other commodity molecules. However, metabolic engineering for increased production of isoprenoids and other bioproducts is limited by an incomplete understanding of factors that control flux through biosynthetic pathways. Here, we examined the native regulation of the isoprenoid biosynthetic pathway in the biofuel producer Zymomonas mobilis. We leveraged oxygen exposure as a means to perturb carbon flux, allowing us to observe the formation and resolution of a metabolic bottleneck in the pathway. Our multi-omics analysis of this perturbation enabled us to identify key auxiliary enzymes whose expression correlates with increased production of isoprenoid precursors, which we propose as potential targets for future metabolic engineering.

## Abstract (precis)

Microbially generated biofuels and bioproducts have the potential to provide a more environmentally sustainable alternative to fossil-fuel-derived products. In particular, isoprenoids, a diverse class of natural products, are chemically suitable for use as high-grade transport fuels and other commodity molecules. However, metabolic engineering for increased production of isoprenoids and other bioproducts is limited by an incomplete understanding of factors that control flux through biosynthetic pathways. Here, we examined the native regulation of the isoprenoid biosynthetic pathway in the biofuel producer Zymomonas mobilis. We leveraged oxygen exposure as a means to perturb carbon flux, allowing us to observe the formation and resolution of a metabolic bottleneck in the pathway. Our multi-omics analysis of this perturbation enabled us to identify key auxiliary enzymes whose expression correlates with increased production of isoprenoid precursors, which we propose as potential targets for future metabolic engineering.

## INTRODUCTION

The mounting costs of fossil fuel extraction and consumption have generated a critical need to develop alternative sources of fuels and commodity molecules. Recently, microbially generated bioproducts have emerged as a promising replacement for fossil-fuel-derived chemicals. Isoprenoids, also called terpenoids, constitute a diverse class of natural products whose members are attractive alternatives to gasoline, diesel, and jet fuel and can also be used as flavor additives, fragrances, pharmaceuticals, and synthetic polymer precursors (1). Metabolic engineering to enhance isoprenoid production in microorganisms has therefore become a growing area of research. Such efforts have focused on two distinct metabolic pathways for isoprenoid biosynthesis: the mevalonate pathway, used in archaea and the cytosol of eukaryotes, and the methyl erythritol 4-phosphate (MEP) pathway, used in most bacteria and in plant chloroplasts. Due to differences in the initial substrates, the MEP pathway has a higher theoretical yield than the mevalonate pathway, making it a more attractive target for metabolic engineering (2). However, knowledge gaps regarding the regulation of MEP pathway activity have prevented well-informed targeted metabolic engineering.

### The MEP pathway: challenges in metabolic engineering.

**FIG 1:**
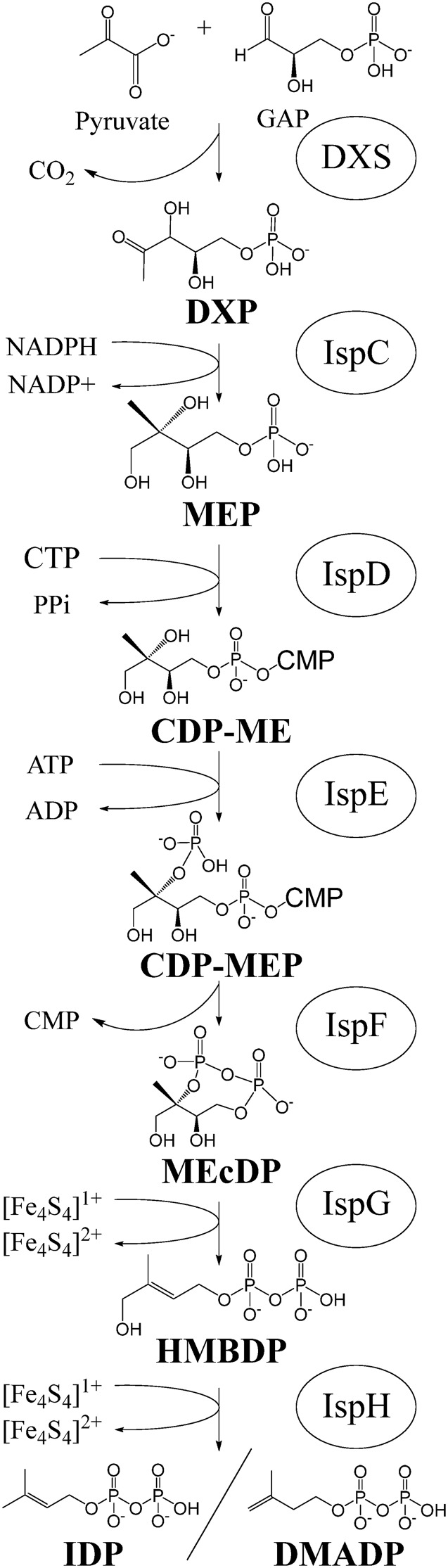
The seven enzymatic steps of the MEP pathway. Intermediates: GAP (d-glyceraldehyde 3-P), DXP (1-deoxy-d-xylulose 5-P), MEP (2-C-methyl-d-erythritol 4-P), CDP-ME (4-diphosphocytidyl-2-C-methyl-d-erythritol), CDP-MEP (4-diphosphocytidyl-2-C-methyl-d-erythritol-2-phosphate), MEcDP (2-C-methyl-d-erythritol 2,4-cylcodiphosphate), HMBDP (4-hydroxy-3-methylbut-2-enyl-diphosphate), DMADP (dimethylallyl diphosphate), IDP (isopentenyl diphosphate). Enzymes: DXS (DXP synthase), IspC (DXS reductoisomerase), IspD (MEP cytidyl transferase), IspE (CDP-ME kinase), IspF (MEcDP synthase), IspG (HMBDP synthase), IspH (HMBDP reductase).

The MEP pathway, depicted in Fig. 1, generates the two five-carbon molecules, isopentenyl diphosphate (IDP) and dimethylallyl diphosphate (DMADP), that are required for the synthesis of all isoprenoids. The final two enzymes of the pathway are IspG, which converts the intermediate 2-C-methyl-d-erythritol 2,4-cyclodiphosphate (MEcDP) to 1-hydroxy-2-methyl-2-(*E*)-butenyl-4-diphosphate (HMBDP), and IspH, which reduces HMBDP to form either IDP or its isomer, DMADP. Both IspG and IspH rely on a [4Fe-4S] cluster cofactor to carry out their reduction reactions (3). Increased expression of IspG and IspH in their apo form is therefore not sufficient to increase enzyme activity, complicating the process of metabolic engineering. Conversion of IspG and IspH to the catalytically active holoenzyme requires construction and insertion of a [4Fe-4S] cluster as well as continual rereduction of the cluster after each catalytic turnover, a process that involves several auxiliary enzymes. As a result, the final two steps of the MEP pathway have become engineering bottlenecks; although several groups have successfully increased production of MEcDP (the substrate of IspG), the production of IDP/DMADP or downstream products has been far more challenging (4–6). For this reason, we have chosen to examine the physiological response to perturbations in MEP pathway with specific emphasis on IspG and IspH.

### Zymomonas mobilis: a promising isoprenoid producer.

The alphaproteobacterium Zymomonas mobilis possesses several desirable characteristics for industrial-scale biofuel production. These include robust anaerobic growth, a broad pH range, and “generally regarded as safe” status (7). Additionally, Z. mobilis exclusively utilizes the highly thermodynamically favorable Entner-Doudoroff (ED) glycolytic pathway, allowing rapid glucose consumption (8). Interest in Z. mobilis as a biofuel producer was initially focused on ethanol production as the species natively converts up to 96% of glucose to ethanol (9). However, this highly catabolic metabolism can also be leveraged for the production of nonethanol products since the carbon natively sent to ethanol can be rerouted toward bioproducts of interest without competing with biomass. Z. mobilis is particularly well suited to production of isoprenoids because the ED pathway, unlike classical glycolysis, generates pyruvate and GAP (d-glyceraldehyde 3-P) in a 1:1 ratio, matching the precursor needs for the MEP pathway. Additionally, Z. mobilis is known to possess lipid membranes containing high levels of hopanoids, a subclass of isoprenoids, suggesting that the species is capable of sustaining high flux through the MEP pathway (10). Despite the potential of Z. mobilis as an isoprenoid-producing organism, very little is known about the regulation of the MEP pathway in this species.

In this study, we employed systems-level approaches to characterize the physiological response to oxygen exposure in Z. mobilis in order to advance our understanding of both the *in vivo* regulation of MEP pathway activity, with specific emphasis on IspG and IspH, and the fundamental physiology of this industrially relevant organism.

## RESULTS

### Experimental design: the oxygen exposure time course.

In order to better understand Z. mobilis physiology, in particular, the metabolic regulation of the MEP pathway, we conducted a systems-level analysis of the response to oxygen exposure. This analysis included quantitation of intracellular and extracellular metabolites by liquid chromatography coupled to mass spectrometry (LC-MS), mRNA sequencing, and shotgun proteomics. We chose to examine oxygen exposure for two main reasons. First, aerobic conditions are known to dramatically change growth and metabolism in Z. mobilis but the precise physiological response to oxygen is still poorly understood (11–13). This response is likely distinct from that of well-studied model organisms due to the unusual highly catabolic metabolism of Z. mobilis. Of particular interest is the fact that Z. mobilis paradoxically contains all the genes required for a functional electron transport chain but exhibits a markedly lower growth rate under aerobic conditions than under anaerobic conditions (14–19). Second, it has been observed in other organisms that oxidative stress impacts MEP pathway activity via inhibition of the [4Fe-4S] cluster cofactors of IspG and IspH (20, 21). The physiological response to oxygen in Z. mobilis is therefore interesting in terms of both characterizing a promising biofuel producer and understanding the regulation of a potential biofuel-producing metabolic pathway.

The oxygen exposure experiment was conducted by first inoculating Z. mobilis ZM4 (ATCC 31821) at a low cell density into minimal media with glucose as the sole carbon source under strict anaerobic conditions. All cultures were kept anaerobic until mid-exponential phase, at which time samples for time point zero were collected for both aerobic (O_2_) and anaerobic (An) cultures. Immediately after time point zero, O_2_ cultures were transferred to aerobic conditions and extractions were taken for metabolomics, transcriptomics, and proteomics analysis at various time points along a 2-h time course. Meanwhile, An cultures, included to control for changes in gene expression and metabolite abundance from mid- to late exponential phase, remained under anaerobic conditions for the duration of the 2-h time course. Both O_2_ and An cultures maintained exponential growth and underwent approximately one doubling during the 2-h time course (see Fig. S1 in the supplemental material).

10.1128/mSystems.00284-18.1 FIG S1 Typical growth of Z. mobilis during the 2-h oxygen exposure time course. Different graphs represent different replicates. Red arrows indicate time of transfer to oxygen. Red circles, cultures transferred to aerobic conditions; blue squares, anaerobic controls. Black arrows indicate *t* = 120 min (final time point). Download FIG S1, TIF file, 0.8 MB. Copyright © 2019 Martien et al. 2019 Martien et al. This content is distributed under the terms of the Creative Commons Attribution 4.0 International license.

### Metabolomics.

**FIG 2:**
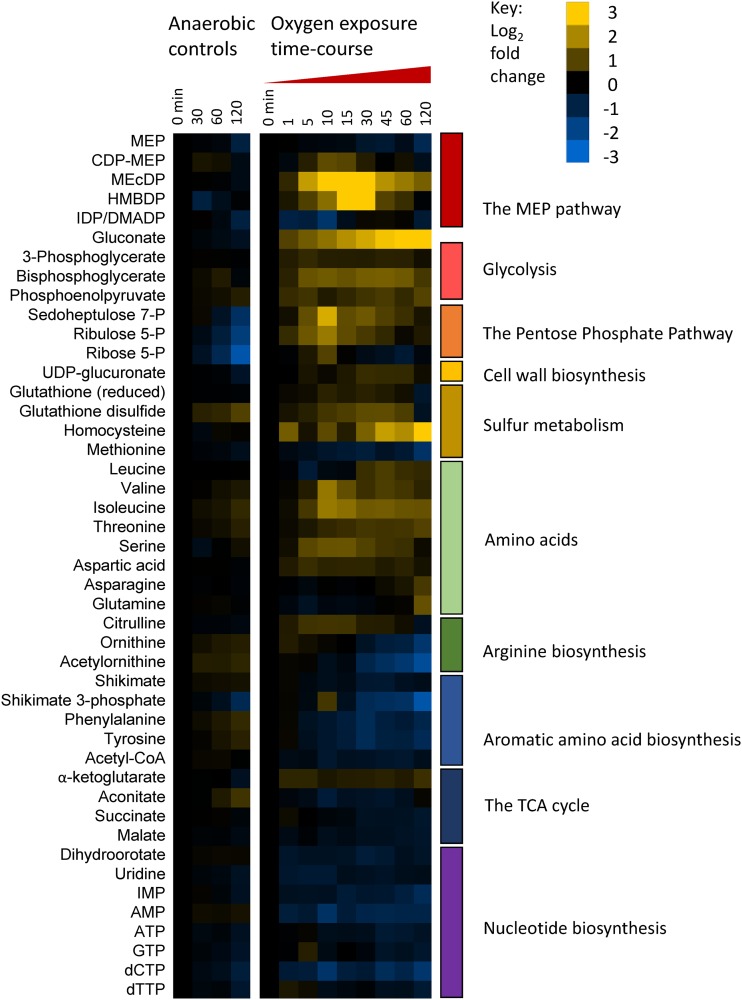
Relative intracellular metabolite levels as detected by LC-MS represented as log_2_ fold change compared to time zero. Yellow indicates increased abundance relative to time zero, blue indicates decreased abundance, and black indicates no change. Anaerobic samples are included to control for metabolic changes that occurred from the mid-exponential phase to the late exponential phase. Metabolites are grouped based on biosynthetic pathway or physiological role. Only metabolites with large and significant changes in response to oxygen are included in this figure (|fold change| > 1.5; FDR < 0.01). Data represent averages of results from at least 3 replicates. For numeric values, *P* values, and FDR data, see Table S2. CoA, coenzyme A.

The LC-MS method used was designed to analyze a broad range of biological compounds and is capable of detecting over 150 metabolites. In this study, we reliably detected 69 metabolites from central carbon metabolism that we could confidently identify based on exact mass and retention time data matched to analytical standards (see Table S1 in the supplemental material). Measured metabolites included intermediates of the ED pathway, the tricarboxylic acid (TCA) cycle, the pentose phosphate pathway (PPP), amino acid biosynthesis, nucleotide biosynthesis, and the MEP pathway. Among the metabolites detected with high confidence, 45 (66%) passed our cutoff criteria of statistically significant and sufficiently large changes in response to oxygen exposure (false-discovery rate [FDR] ≤ 0.01, |fold change| [FC] ≥ 1.5) (Fig. 2). Some of the largest changes observed include alterations in the levels of MEP pathway intermediates; accumulation of gluconate; a temporary spike in intermediates of the PPP; perturbations in sulfur metabolism, branched-chain amino acid biosynthesis, and arginine biosynthesis; and depletion of aromatic amino acids and nucleotides (Fig. 2).

10.1128/mSystems.00284-18.8 TABLE S1 Average log_2_ fold change values for all detected metabolites. “NA” indicates that signal intensity was too low to obtain reliable data at that time point (below 1−e4). Columns represent condition/time points; rows represent metabolites. The final two columns show *P* values and false-discovery rates for each metabolite over the oxygen exposure time course (O_2_ samples) using repeated-measures ANOVA. An, anaerobic control samples. Download Table S1, XLSX file, 0.02 MB. Copyright © 2019 Martien et al. 2019 Martien et al. This content is distributed under the terms of the Creative Commons Attribution 4.0 International license.

10.1128/mSystems.00284-18.9 TABLE S2 Average log_2_ fold change values of protein and mRNA abundances for all detected genes and proteins. “NA” indicates that no data were obtained for a given gene. Columns represent condition/time points; rows represent genes. Genes are ordered by locus tag. The columns in the first set (3rd to 13th) were obtained from the proteomics data. The columns in the second set (14th to 25th) were obtained from mRNA sequencing. The final two columns in each set show *P* values and false-discovery rates for each gene over the oxygen exposure time course (O_2_ samples) as determined using repeated-measures ANOVA. An, anaerobic control samples. Download Table S2, XLSX file, 0.5 MB. Copyright © 2019 Martien et al. 2019 Martien et al. This content is distributed under the terms of the Creative Commons Attribution 4.0 International license.

### A temporary metabolic bottleneck in isoprenoid biosynthesis.

**FIG 3:**
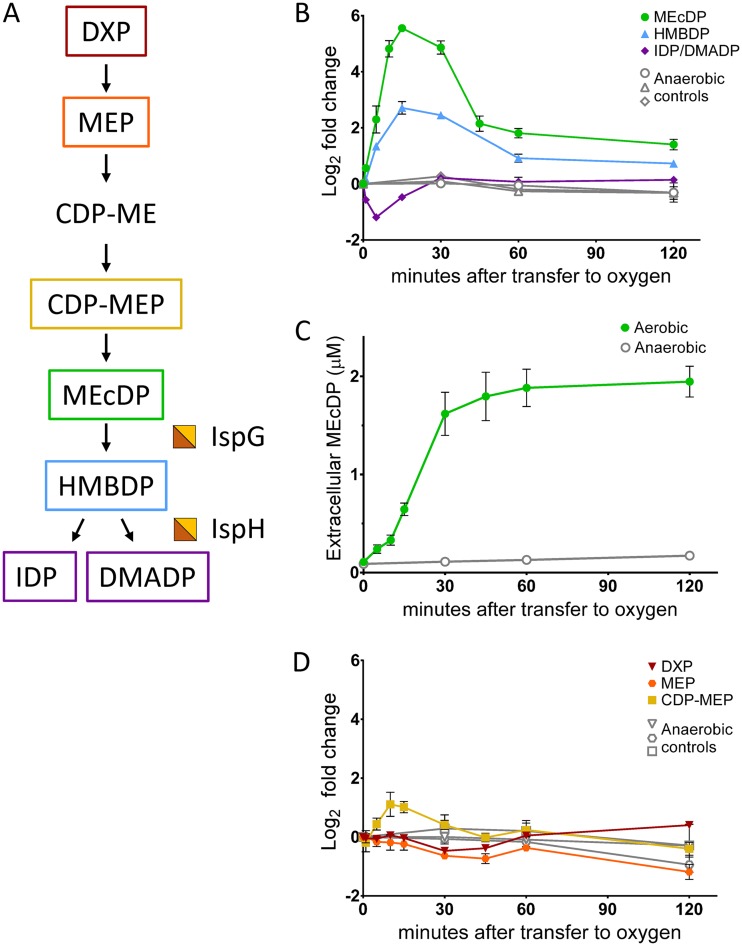
Intermediates of the MEP pathway during exposure to oxygen. (A) Simplified schematic of the MEP pathway. Half-orange, half-yellow squares represent the iron-sulfur cluster cofactors of IspG and IpsH. (B, C, and D) Filled, cultures transferred to aerobic conditions immediately after time zero; open, anaerobic controls. Data represent averages of results from at least three replicates. Error bars show standard errors of the means (SEM). For points with no visible error bars, the bars are within the size of the symbol. (B and D) Relative intracellular metabolite concentrations of the intermediates of the MEP pathway, represented as log_2_ fold change relative to time zero. (B) Green circles, MEcDP (2-C-methyl-d-erythritol 2,4-cylcodiphosphate); blue triangles, HMBDP (4-hydroxy-3-methylbut-2-enyl-diphosphate); purple diamonds, isomers IDP (isopentenyl diphosphate) and DMADP (dimethylallyl diphosphate), not resolvable by our LC-MS methods and therefore represented as their combined values. (C) Absolute extracellular concentrations of MEcDP. (D) Red upside-down triangles, DXP (1-deoxy-d-xylulose 5-P); orange circles, MEP (2-C-methyl-d-erythritol 4-P); yellow squares, CDP-ME (4-diphosphocytidyl-2-C-methyl-d-erythritol).

Of all measured metabolites, the metabolite that displayed the most dramatic response to oxygen exposure was MEcDP, the fifth intermediate of the MEP pathway (Fig. 3A and B). Intracellular MEcDP levels displayed a transient accumulation, reaching their peak after 15 min of oxygen exposure and returning to near-baseline levels by 60 min (Fig. 3B). The maximum change compared to the anaerobic zero time point was a 45-fold increase. By 120 min, MEcDP levels had dropped back to within a 3-fold increase. MEcDP also accumulated extracellularly, reaching a concentration of 2 µM by 30 min, but did not return to baseline levels when intracellular levels began to drop (Fig. 3C). MEcDP was the only intermediate of the MEP pathway that could be detected extracellularly.

Accompanying the increase in MEcDP levels was a less severe but temporally corresponding accumulation of HMBDP, the intermediate immediately downstream of MEcDP. Intracellular HMBDP levels also peaked at 15 min, reaching a 6.5-fold increase, and returned to within 2-fold of baseline by 60 min (Fig. 3B). The intermediates immediately downstream of HMBDP, isomers IDP and DMADP, were detected as a single signal using our LC-MS methods. Their combined levels also changed transiently but in the opposite direction compared to MEcDP and HMBPP. Levels of IDP/DMADP reached a 2-fold decrease after 5 min of oxygen exposure and returned to baseline levels by 30 min, corresponding to the time when MEcDP and HMBDP levels began decreasing (Fig. 3B). Intermediates of the MEP pathway upstream of MEcDP displayed a limited response to oxygen; 4-diphosphocytidyl-2-C-methyl-d-erythritol-2-phosphate (CDP-MEP) accumulated around 2-fold between 10 and 15 min, but MEP and 1-deoxy-d-xylulose 5-phosphate (DXP) showed little to no change (Fig. 3D).

The observed changes in intracellular levels of MEP pathway intermediates upon exposure to oxygen were indicative of a short-term metabolic bottleneck in the lower MEP pathway that temporarily blocked carbon flow from MEcDP to IDP/DMADP. To confirm inhibition of carbon incorporation into IDP/DMADP, we repeated the oxygen shift experiment, this time adding universally labeled [^13^C]glucose to Z. mobilis cultures 7 min after the transfer to oxygen (estimated to be the time of maximal IspG/H inhibition based on the data shown in Fig. 3B). Consistent with oxygen-induced inactivation of the FeS enzymes IspG and IpsH, ^13^C carbon incorporation into DXP and MEcDP was not severely inhibited by oxygen exposure. However, the rate of ^13^C carbon incorporation into IDP/DMADP was at least an order of magnitude lower in the culture transferred to oxygen than in the anaerobic control (Fig. S2).

10.1128/mSystems.00284-18.2 FIG S2 Intracellular levels of ^13^C-labeled (orange circles) or ^12^C-unlabeled (blue squares) intermediates of the MEP pathway either under anaerobic conditions (left) or after transfer to aerobic conditions (right). *x*-axis values show minutes after transfer to oxygen. The red dotted line indicates time of ^13^C addition. *y*-axis values show raw signal intensities detected via LC-MS. Due to carbon rearrangements, multiple labeled forms (i.e., labeled at 2, 3, or all 5 carbons) of each metabolite existed during growth on a mixture of ^12^C and ^13^C carbon. Here, all forms that included at least 2 ^13^C-labeled carbons were combined to generate the orange data points. Data are from a single replicate for each condition. (A) Deoxy-xylulose 5-phosphate (DXP). (B) 2-C-methyl-d-erythritol 2,4-cylcodiphosphate (MEcDP). The anaerobic controls (green triangles) are included in the aerobic graph for comparison in the same scale. (C) The combined signal from the isopentenyl pyrophosphate and dimethylallyl pyrophosphate (IDP/DMADP) isomers. Download FIG S2, TIF file, 1.1 MB. Copyright © 2019 Martien et al. 2019 Martien et al. This content is distributed under the terms of the Creative Commons Attribution 4.0 International license.

The metabolic bottleneck in the lower MEP pathway was likely caused by oxidative damage to the [4Fe-4S] cluster cofactors of IspG and IspH; the sensitivity of IspG and IspH to oxidative stress is well documented in bacteria and in plants as well as with purified enzyme (20–23). To confirm this, we exposed Z. mobilis to cadmium (CdCl_2_) under anaerobic conditions as a second form of [4Fe-4S] stress. Cadmium can be incorporated into FeS clusters in place of iron, disrupting the activity of FeS enzymes (24). We observed dramatic accumulation of MEcDP and depletion of IDP/DMADP upon treatment with CdCl_2_, supporting the conclusion that FeS damage is the driving force behind the bottleneck in the MEP pathway during exposure to oxygen (Fig. S3A and B).

10.1128/mSystems.00284-18.3 FIG S3 Relative intracellular metabolite levels for (A) 2-C-methyl-d-erythritol 2,4-cylcodiphosphate (MEcDP) and (B) combined levels of isopentenyl diphosphate and dimethylallyl diphosphate (IDP/DMADP) and (C) gluconate following treatment with 0.15 mM (left) or 0.05 mM (right) Cd^2+^. Data are represented as log_2_ fold change relative to time zero (before addition of Cd^2+^). Data represent averages of results from two replicates, except for the 120-min time point for 0.15 mM Cd^2+^, which presents data from only one replicate due to a technical error. Error bars show SEM. For points with no visible error bars, the bars are within the size of the symbol (except for the 120-min time point for 0.15 mM). Red circles, cultures exposed to Cd^2+^; black squares, controls (not exposed to Cd^2+^). Download FIG S3, TIF file, 0.9 MB. Copyright © 2019 Martien et al. 2019 Martien et al. This content is distributed under the terms of the Creative Commons Attribution 4.0 International license.

### Transcriptomics and proteomics.

**FIG 4:**
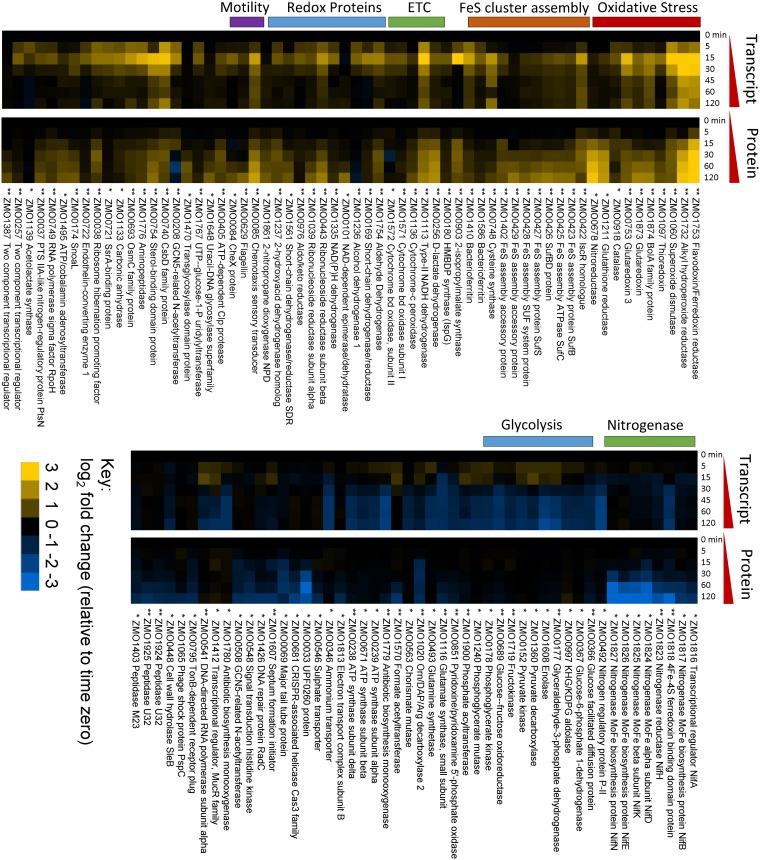
Relative mRNA transcript and protein levels during oxygen exposure represented as log_2_ fold change relative to time zero. Yellow indicates increased expression relative to time zero, blue indicates a decrease, and black indicates no change. Rows are aligned to represent transcript and protein level for a single gene. Genes are organized by function and relative expression levels. Upregulated genes are on the left and downregulated genes are on the right. Only annotated genes with large and significant changes in response to oxygen at either the transcript or the protein level are represented here (|fold change| > 2; FDR < 0.01). Asterisks (*) indicate statistical significance; an asterisk on the left indicates significance at the transcript level and an asterisk on the right indicates significance at the protein level. Significantly changing unannotated genes and ribosomal protein genes are represented in Fig. S4. Data represent averages of results from at least three replicates. For numeric values, *P* values, FDR, and anaerobic controls, see Table S3. ETC, electron transport chain.

In order to better understand the physiological mechanism by which Z. mobilis recovers from the metabolic bottleneck induced by oxygen exposure, leading to the return to baseline levels of MEcDP, HMBDP, and IDP/DMADP, we conducted transcriptomic and proteomic profiling along the same oxygen exposure time course. We obtained relative abundances of both protein and mRNA transcripts for 1,565 of 1,890 protein-coding genes in the ZM4 genome, corresponding to 82.8% coverage (Table S2). Our analysis identified 166 genes (10% of the detected genome) that changed significantly in either transcript or protein abundance in response to oxygen (FDR ≤ 0.01 and FC ≥ 2) (Fig. 4; see also Fig. S4).

10.1128/mSystems.00284-18.4 FIG S4 Relative mRNA transcript and protein levels during oxygen exposure represented as log_2_ fold change relative to time zero. Yellow indicates increased expression relative to time zero, blue indicates decreased expression, and black indicates no change. Rows are aligned to represent transcript and protein levels for a single gene. Genes are organized by location in the genome. Only genes with large and significant changes in response to oxygen are represented here (|fold change| > 2; FDR < 0.01). Ribosomal protein genes are on the left; uncharacterized genes are on the right. Download FIG S4, TIF file, 1.2 MB. Copyright © 2019 Martien et al. 2019 Martien et al. This content is distributed under the terms of the Creative Commons Attribution 4.0 International license.

10.1128/mSystems.00284-18.10 TABLE S3 Schematic of sampling effort for the four iterations of the oxygen exposure time course experiment whose results were averaged together to generate log_2_ values for metabolomics data. Time after transfer to oxygen is represented in minutes. The red outline indicates an extraction event for aerobic samples. The blue outline indicates an extraction event for anaerobic controls. Yellow fill indicates extraction for metabolomics analysis. Green fill indicates extraction for proteomics analysis. Pink fill indicates extraction for mRNA sequencing. The number in each box indicates the number of replicates for that extraction type at that time point. Download Table S3, XLSX file, 0.01 MB. Copyright © 2019 Martien et al. 2019 Martien et al. This content is distributed under the terms of the Creative Commons Attribution 4.0 International license.

Several known oxidative stress response genes were found to be upregulated upon exposure to oxygen, including superoxide dismutase, alkylhydroperoxide reductase, and glutathione reductase genes (Fig. 4) (25). Additionally, we observed upregulation of genes involved with iron-sulfur cluster biogenesis and maintenance, the electron transport chain, redox proteins, and chemotaxis. The most dramatic downregulation was observed in levels of nitrogenase proteins, known to be inactivated by oxygen (26). There was excellent agreement between mRNA transcript levels and the protein level for upregulated genes (Fig. 4). Upregulated genes tended to display elevated transcript levels first, followed by an increase in protein levels, as expected given the necessity of mRNA transcript for translation. Strikingly, the peak in mRNA levels consistently occurred 15 min after oxygen exposure for upregulated genes, indicating a concerted and acute response to oxygen that was primarily transcriptionally driven (Fig. 4). Many downregulated genes displayed a steady decrease in protein levels but little to no change in transcript levels, consistent with protein degradation rather than transcriptional control (Fig. 4). Indeed, protease-dependent degradation of cyanobacterial nitrogenase during exposure to oxygen has been previously reported (27). Other genes were downregulated at the transcriptional level but displayed no change at the protein level, perhaps reflective of the long half-life of protein compared to mRNA.

### Iron-sulfur cluster auxiliary proteins.

**FIG 5:**
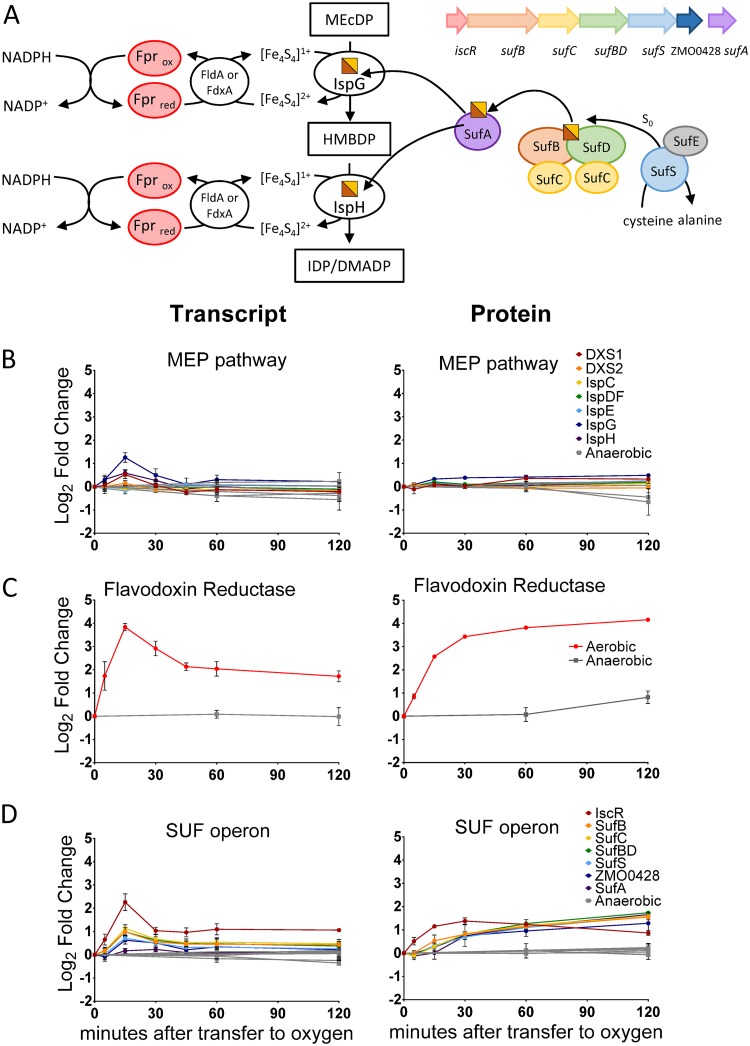
(A) Schematic of the assembly and reduction of the [Fe_4_S_4_] cluster cofactors of IspG and IspH. The orientation, arrangement, and approximate size of the genes in the Z. mobilis *suf* operon are indicated. (B, C, and D) Relative levels of mRNA transcript (left) and protein (right) represented as log_2_ fold change relative to time zero. All graphs use the same axis units. Colored circles, cultures transferred to aerobic conditions; gray squares, anaerobic controls. Data represent averages of results from at least three replicates. Error bars show standard errors of the means. For points with no visible error bars, the bars are within the size of the symbol. (B) Enzymes of the MEP pathway, including two DXS homologues and the bi-functional IspDF enzyme. (C) Flavodoxin reductase. (D) Enzymes of the *suf* operon.

Despite the dramatic decrease in MEcDP and HMBDP levels between 15 and 60 min after oxygen exposure, expression of the enzymes in the MEP pathway remained relatively constant throughout the time course. Protein levels of all MEP pathway enzymes remained within 1.5-fold of the anaerobic baseline (Fig. 5B). Only IspG displayed a fold change in mRNA transcript levels above 1.5, reaching a 2.38-fold increase after 15 min of oxygen exposure (Fig. 5B). Although increased expression of IspG may contribute to the resolved bottleneck in the MEP pathway, it would be unexpected for such a small change in the protein level alone to bring about the fast recovery of IspG and IspH enzymatic activity evidenced by the metabolomics data. Our transcriptomics and proteomics data indicate instead that auxiliary proteins involved in FeS cluster maintenance and biogenesis play a critical role in MEP pathway recovery.

The largest increase in protein abundance—and the second largest increase in transcript abundance—corresponded to flavodoxin/ferredoxin reductase (Fpr), whose abundance was increased by over 17-fold in protein level and 14-fold in transcript level during the oxygen exposure time course (Fig. 5C). Fpr was also among the first proteins to respond to oxygen, together with superoxide dismutase and hydroperoxide reductase. Fpr is of special significance with respect to the MEP pathway as it catalyzes the transfer of electrons from NADPH to flavodoxin (FldA) and ferredoxin (FdxA) (Fig. 5A). Both FldA and FdxA have been demonstrated previously to deliver electrons to the FeS clusters of IspG and IspH. The use of FldA or FdxA depends on the organism (FldA in Escherichiacoli; FdxA in Arabidopsis thaliana and Plasmodium falciparum) (28–30). It is unknown whether Z. mobilis utilizes FldA or FdxA as the electron shuttle between Fpr and IspG/H. However, in either case, Fpr is required to deliver electrons from NADPH to the IspG/H-reducing enzyme. Neither FldA nor FdxA was found to increase in abundance in response to oxygen (FDR > 0.02, fold increase < 1.5).

During exposure to oxygen, hydrogen peroxide and superoxide can overoxidize [4Fe-4S] clusters, forming the [4Fe-4S]^3+^ state, which is chemically unstable and readily releases an Fe^2+^ ion, leaving behind the catalytically inactive [3Fe-4S]^1+^ partial cluster (31). IspG and IspH are especially susceptible to oxidative damage by univalent oxidants because the catalytically active iron in the cluster is solvent exposed rather than coordinated by a cysteine residue (3). Upregulation of Fpr likely helps restore MEP pathway activity by maintaining IspG and IspH in their fully reduced [4Fe-4S]^1+^ state, potentially both increasing catalytic turnover and protecting FeS clusters from overoxidation and Fe^2+^ release.

Those clusters that are damaged by overoxidation must be repaired or replaced. Replacement requires *de novo* FeS cluster assembly, which, in Z. mobilis, is carried out by the *suf* operon, the only [4Fe-4S] cluster biogenesis operon in its genome (Fig. 5A) (32, 33). The *suf* operon was also upregulated during the response to oxygen. Upregulation of all seven genes in this operon were found to be statistically significant (FDR < 0.01) at both the protein and transcript levels (Fig. 5D). Interestingly, the first gene in the *suf* operon, ZMO0422 (a homologue of the transcriptional regulator IscR), increased in transcript and protein levels before the downstream genes, whose increased expression occurred after a short delay. After 5 min of oxygen exposure, ZMO0422 exhibited a 1.5-fold increase in both mRNA and protein levels whereas expression levels of the rest of the operon remained within the error range of anaerobic baseline levels (Fig. 5D). Additionally, the levels of ZMO0422 protein peaked after 30 min of oxygen exposure, whereas the levels of the remaining *suf* operon proteins continued to increase throughout the time course, albeit at a decreasing rate (Fig. 5D). These trends suggest that the Z. mobilis IscR homologue increases transcription of the *suf* operon during oxidative stress, consistent with what has been observed in E. coli (34). However, the mechanism of increased transcription (i.e., activation or derepression) may be different in the two species. In general, the genes of the *suf* operon exhibited concerted expression levels for both mRNA and protein, as would be expected from a polycistronic operon being cotranscriptionally translated. The consistency with respect to protein level between genes of the same operon also highlights the fact that this response appears to transcriptionally regulated.

Together, upregulation of Fpr and the *suf* operon likely increases the enzymatic activity of IspG and IspH by ensuring that their FeS cofactors are intact and in the reduced [4Fe-4S]^1+^ state. The temporal patterns of MEcDP accumulation and gene expression support this hypothesis; both the Frp gene and the *suf* operon genes approached a new, elevated, steady-state level after 30 min of oxygen exposure, corresponding to the time when MEcDP levels began to drop. The fact that levels of MEP pathway enzymes changed only marginally throughout the time course highlights the importance of cofactor-maintaining auxiliary enzymes in regulating MEP pathway activity.

### Other metabolic changes related to iron-sulfur clusters.

IspG and IspH are just two among several enzymes in central carbon metabolism that rely on FeS clusters to catalyze enzymatic reactions. Various enzymes involved in the TCA cycle, amino acid biosynthesis, and the ED pathway are also known to contain oxygen-sensitive [4Fe-4S] cofactors (35). In particular, members of the hydro-lyase class of enzyme often contain [4Fe-4S] clusters that, like IspG and IspH, include one solvent-exposed iron, rendering them especially susceptible to oxidative damage (31). Consistent with our conclusion that MEcDP accumulation is driven by FeS cluster damage, we observed changes in the levels of products and substrates of other oxygen-sensitive FeS enzymes and of metabolites required for FeS cluster biogenesis.

**FIG 6:**
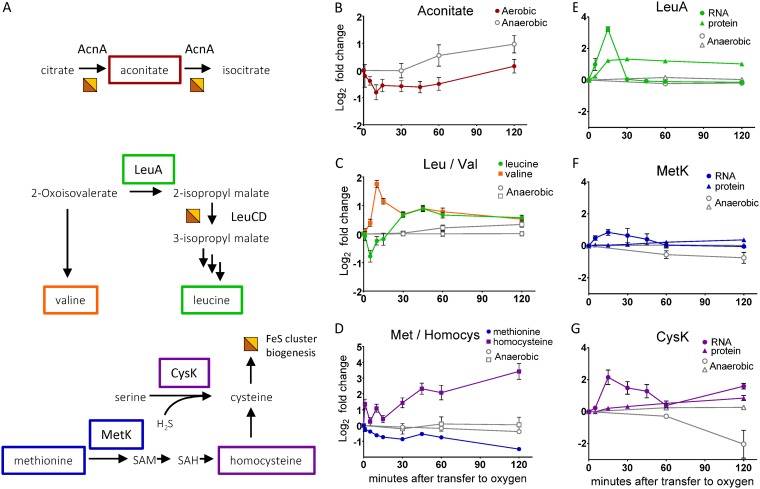
(A) Schematic of iron-sulfur cluster enzymes and biogenesis. Half-orange, half-yellow squares next to an enzymatic reaction indicate that the enzyme utilizes an iron-sulfur cluster cofactor. A half-orange, half-yellow square as the product of an enzymatic reaction represents iron-sulfur biogenesis. Abbreviations: SAM, S-adenosylmethionine; SAH, S-adenosylhomocysteine. (B, C, D, E, F, and G) Filled symbols, cultures transferred to aerobic conditions immediately after time zero; open symbols, anaerobic controls. Data represent averages of results from at least three replicates. Error bars show SEM. For points with no visible error bars, the bars are within the size of the symbol. (B, C, and D) Relative intracellular metabolite concentrations of metabolites related to iron-sulfur clusters represented as log_2_ fold change relative to time zero. (B) Levels of aconitate. (C) Levels of leucine (green circles) and valine (orange squares). (D) Levels of methionine (blue circles) and homocysteine (purple squares). (E, F, and G) Relative levels of mRNA transcript (circles) and protein (triangles) represented as log_2_ fold change relative to time zero. (E) Isopropyl malate synthase (LeuA) transcript and protein level. (F) S-adenosylmethionine synthetase (MetK) transcript and protein level. (G) Cysteine synthase (CysK) transcript and protein level.

Aconitate hydratase (AcnA), the TCA cycle enzyme that converts citrate to isocitrate via the intermediate aconitate, is a well-studied hydro-lyase that contains a [4Fe-4S] cofactor and is known to be sensitive to oxidative damage (Fig. 6A) (36). Consistent with oxygen-induced inactivation of AcnA, intracellular levels of aconitate displayed a transient decrease following the transfer to oxygen, similarly to what was observed for IDP/DMADP (Fig. 6B).

Another [4Fe-4S]-dependent hydro-lyase, isopropylmalate isomerase (LeuCD), converts 2-isopropylmalate to 3-isopropylmalate, thereby directing carbon away from valine and toward leucine biosynthesis (Fig. 6A) (37). This pathway is of particular relevance to bioproduct generation as the 2-ketoacid intermediates of branched-chain amino acid biosynthesis can be converted to valuable higher alcohols such as isobutanol (38). Consistent with transient oxidative damage to the FeS cluster of LeuCD, leucine levels dropped in response to oxygen exposure, while valine levels temporarily increased (Fig. 6C). Leucine levels reached their minimum 5 min after transfer to aerobic conditions, and valine levels peaked after 15 min, temporally consistent with the drop and spike in IDP/DMADP and MEcDP levels, respectively (compare Fig. 3B to Fig. 6C). This bottleneck was resolved with no significant changes in the expression level of the LeuCD enzyme (FRD > 0.01, FC < 1.2). However, we did observe a significant increase in transcript and protein abundance for 2-isopropylmalate synthase (LeuA), the enzyme directly upstream of LeuCD (Fig. 6E). Upregulation of LeuA is likely one factor, in addition to upregulation of the *suf* operon, that allowed the return of both leucine and valine to near-baseline levels.

In addition to carbon metabolism, we observed changes in sulfur metabolism that may be explained by oxidative damage to FeS clusters. During FeS cluster repair and *de novo* biogenesis, cysteine serves as the source of reduced sulfur (39). Although our LC-MS methods were unable to reliably detect cysteine, we did observe continuous depletion of methionine together with accumulation of homocysteine along the oxygen exposure time course (Fig. 6D). These trends are consistent with a conversion of methionine to cysteine as a means to keep up with elevated demands for cysteine due to FeS cluster damage. Increased transcript and protein levels for cysteine synthase (CysK) further support this conclusion. Although the increase in S-adenosylmethionine synthetase (MetK) mRNA and protein levels did not pass our FDR threshold for statistical significance (FDR = 0.045 and 0.0102, respectively), they followed the same trend as CysK, indicating that both paths to cysteine, from methionine via homocysteine and from serine via CysK, may be upregulated during oxygen exposure (Fig. 6F and G). Methionine depletion may be further driven by oxidative inactivation of cobalamin-independent methionine synthase (MetE), as has been observed in both E. coli and Bacillus subtilis (40–42). The importance of increased cysteine production following exposure to oxygen is further highlighted by the fact that supplementation of minimal media with cysteine but not serine improved aerobic growth of Z. mobilis (Fig. S5).

10.1128/mSystems.00284-18.5 FIG S5 Z. mobilis growth after transfer to oxygen at a low cell density in minimal media (blue squares), minimal media plus 0.5 mM cysteine (yellow circles), or minimal media plus 0.5 mM serine (red triangles). The red arrow indicates time of transfer to aerobic conditions. Growth was measured by measuring optical density at 600 nm. Download FIG S5, TIF file, 0.5 MB. Copyright © 2019 Martien et al. 2019 Martien et al. This content is distributed under the terms of the Creative Commons Attribution 4.0 International license.

### Gluconate production under aerobic conditions.

**FIG 7:**
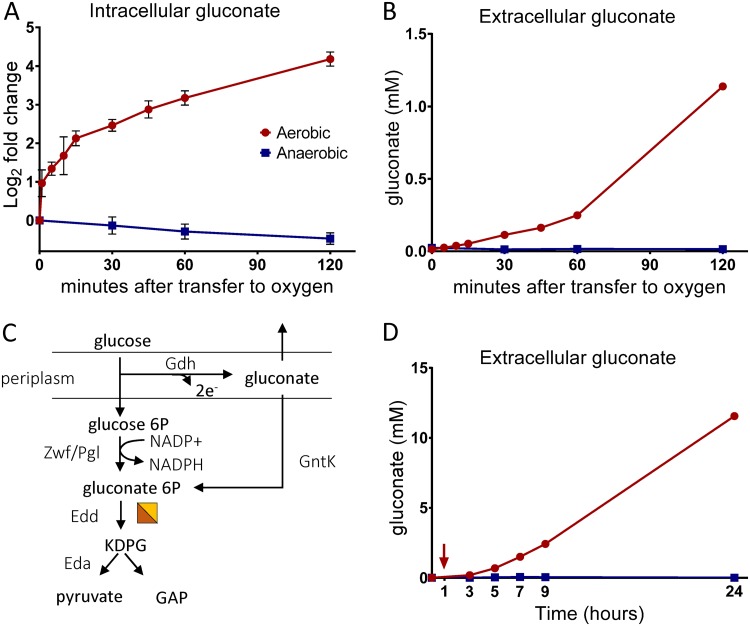
(A, B, and D) Gluconate levels as measured by LC-MS. Red circles, cultures transferred to aerobic conditions; blue squares, anaerobic controls. Data represent averages of results from at least three replicates. Error bars show SEM. For points with no visible error bars, the bars are within the size of the symbol. (A) Relative intracellular levels of gluconate represented as log_2_ fold change relative to time zero. (B) Absolute concentration of extracellular gluconate during a 2-h time course following transfer to aerobic conditions. (C) Schematic of the oxidation of glucose to gluconate and the upper ED pathway. The half-orange half-yellow square indicates an FeS-dependent enzyme. Abbreviations: Gdh, glucose dehydrogenase; GntK, gluconate kinase; Zwf, glucose-6-phosphate 1-dehydrogenase; Pgl, 6-phosphogluconolactonase; Edd, phosphogluconate dehydratase; Eda, KDPG aldolase; KDPG, 2-dehydro-3-deoxy-phosphogluconate; GAP, glyceraldehyde 3-phosphate. (D) Absolute concentration of extracellular gluconate during a 24-h time course. The red arrow indicates the time of transfer to aerobic conditions.

After MEcDP, the second most dramatic metabolic change observed along the oxygen exposure time course was in intracellular gluconate levels, which increased by 18-fold above the anaerobic baseline (Fig. 7A). Gluconate also accumulated extracellularly, reaching concentrations above 1 mM after 2 h of aerobic growth (Fig. 7B). Unlike the MEcDP levels, gluconate levels continued to increase throughout the 2-h time course and did not return to baseline. Gluconate accumulation may have been the result of FeS cluster damage, as the ED pathway enzyme 6-phosphogluconate dehydratase (Edd) contains a [4Fe-4S] cofactor (Fig. 7C). The trends seen in intracellular metabolite levels of upper ED pathway intermediates tend to support this conclusion; glucose 6-phosphate and 6-phosphogluconate increased in abundance whereas 2-dehydro-3-deoxy-phosphogluconate (KDPG) decreased in abundance immediately after transfer to oxygen (Fig. S6A to C). However, none of upper ED pathway intermediates displayed a change of greater than 1.5-fold and all three returned to baseline levels after 2 h. Therefore, the observed continual and dramatic accumulation of gluconate does not seem to be wholly explained by inactivation of Edd. Furthermore, when Z. mobilis was subjected to nonoxidative FeS stress by exposure to cadmium under anaerobic conditions, gluconate did not accumulate (Fig. S3C). These observations suggest that gluconate accumulation is driven by a regulatory response to oxygen in addition to acute inhibition of Edd.

10.1128/mSystems.00284-18.6 FIG S6 Relative intracellular metabolite concentrations, represented as log_2_ fold change relative to time zero. Error bars show SEM. For points with no visible error bars, the bars are within the size of the symbol. Red circles, cultures transferred to aerobic conditions at time zero; blue squares, anaerobic controls. (A to C) Levels of the first three intermediates of the Entner-Doudoroff glycolytic pathway. KDPG, 2-dehydro-3-deoxy-phosphogluconate. (D) Levels of NADH. Download FIG S6, TIF file, 0.8 MB. Copyright © 2019 Martien et al. 2019 Martien et al. This content is distributed under the terms of the Creative Commons Attribution 4.0 International license.

**FIG 8:**
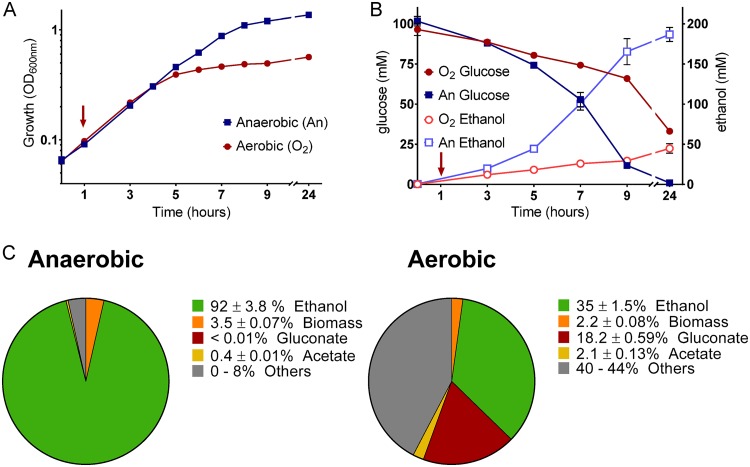
(A and B) Growth, glucose consumption, and ethanol production of Z. mobilis under strict anaerobic conditions (blue squares) or after transfer to aerobic conditions at a low cell density (red circles). The red arrow indicates the time of transfer to aerobic conditions. Data represent averages of results from three replicates. Error bars show standard errors of the means. For points with no visible error bars, the bars are within the size of the symbol. (A) Growth measured as optical density at 600 nm. (B) Glucose consumption measured by LC-MS (solid symbols) and ethanol production measured by NMR (open symbols). (C) Carbon utilization by Z. mobilis under strict anaerobic conditions (left) or after transfer to aerobic conditions at a low cell density (right). Values represent averages of results from three replicates ± SEM. Percentages were calculated based on the molar potential of given biosynthetic pathways (e.g., 1 mol glucose generates 2 mol ethanol). CO_2_ production data are not included. Lactate, shikimate, and succinate levels were also measured but accounted for less than 0.1% of glucose consumption under both sets of conditions. Biomass values were calculated using 0.41 g of cell dry weight per liter·OD_600_ (optical density at 600 nm) and 48% carbon in cell dry weight.

To better understand this long-term metabolic response, we examined extracellular metabolite concentrations along a 24-h time course during which Z. mobilis was transferred to aerobic conditions at a low cell density (Fig. 8A). In addition to LC-MS analysis, we quantified extracellular ethanol and acetate levels by nuclear magnetic resonance (NMR) analysis during this time course. As has been previously reported, we found that the presence of oxygen was inhibitory to both growth and ethanol production in Z. mobilis (Fig. 8) (12, 13). Ethanol production accounted for 92.6% of the glucose consumed for anaerobic cultures, compared to only 35% for aerobic cultures, at the end of the 24-h time course (Fig. 8C). Decreased ethanol production was accompanied by an increase in production of gluconate, which was continually excreted over the 24-h time course under aerobic conditions, following the trends observed in the 2-h time course (Fig. 7D). The final extracellular gluconate concentration was 11.5 mM, representing 18% of the glucose consumed (Fig. 8C). Anaerobic cultures did not accumulate gluconate to any appreciable extent (<0.01% of glucose was converted to gluconate). There was also an increase in the extracellular levels of acetate and pyruvate under aerobic conditions (Fig. 8C; see also Fig. S7). However, the concentrations of nonethanol products did not account for the large discrepancy between aerobic and anaerobic conditions with respect to ethanol production; 42% of the glucose consumed by aerobically grown cultures did not correspond to the metabolites that we detected (Fig. 8C). It is possible that some fraction of this missing carbon evaporated as acetaldehyde given that acetaldehyde production by aerobically growing Z. mobilis has been previously reported and that our culturing conditions and sample preparation were not optimized for highly volatile compounds (16, 43).

10.1128/mSystems.00284-18.7 FIG S7 Extracellular metabolite levels during anaerobic growth (blue squares) or after transfer to oxygen at a low cell density (red circles). The red arrow indicates the time of transfer to oxygen. (Left) Pyruvate, measured by LC-MS. (Right) Acetate, measured by NMR. Download FIG S7, TIF file, 0.1 MB. Copyright © 2019 Martien et al. 2019 Martien et al. This content is distributed under the terms of the Creative Commons Attribution 4.0 International license.

The changes observed in the extracellular metabolite profile under aerobic conditions are consistent with an oxygen-induced shift in redox balancing. The dramatic decrease in ethanol production under aerobic conditions may be explained by deceased availability of NADH resulting from its utilization by competing reactions (Fig. S6D). Additionally, the conversion of glucose to gluconate generates reducing power which must inevitably influence the redox balance of the cell. On the basis of the transcriptomics and proteomics data, the noncanonical Z. mobilis electron transport chain may be responsible for the increased NADH consumption and may also be the fate of electrons from the oxidation of glucose.

### Upregulation of the electron transport chain during aerobic growth.

**FIG 9:**
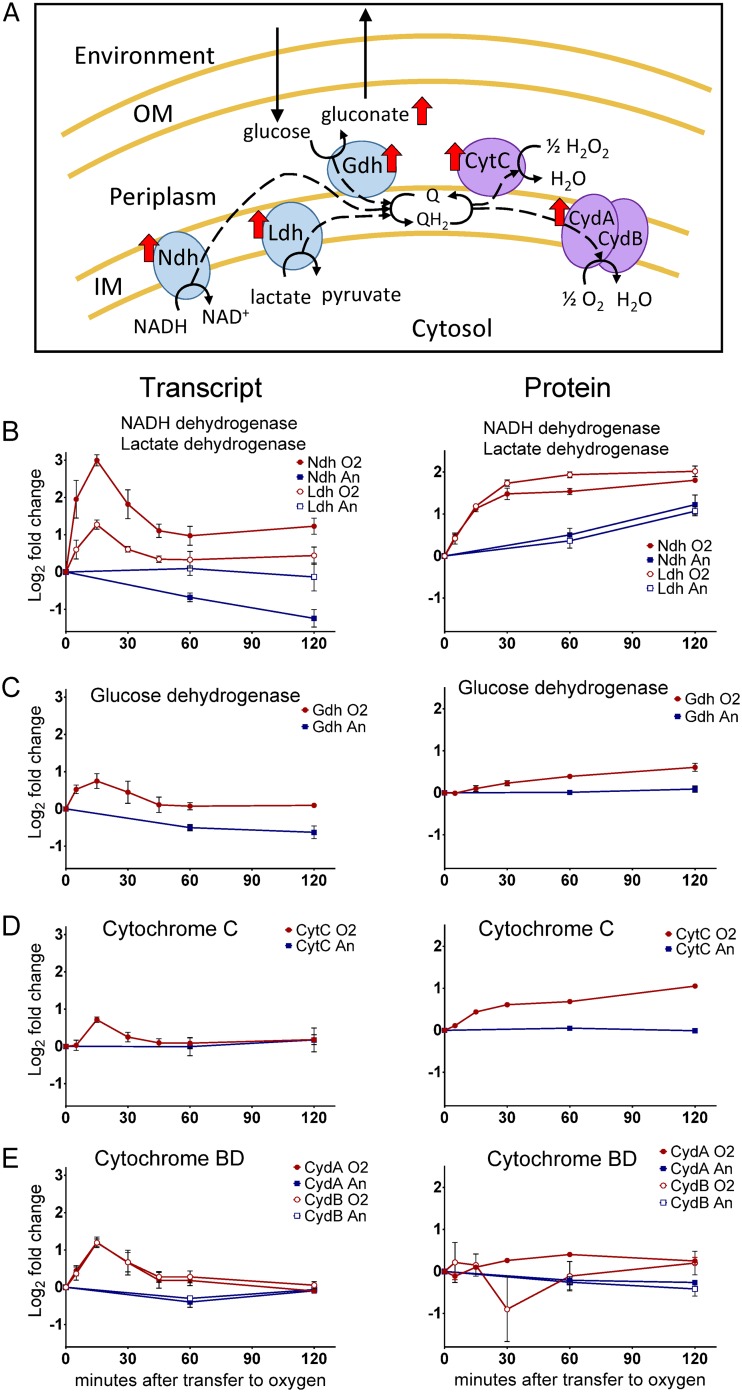
(A) Diagram of the Z. mobilis electron transport chain. Red arrows next to a protein or metabolite indicate upregulation in response to oxygen. IM, inner membrane; OM, outer membrane. Positioning based on predicted localization using psortb v.3.0 (www.psort.org). (B, C, D, and E) Red circles, cultures transferred to aerobic conditions; blue squares, anaerobic controls. Relative levels of mRNA transcript (left) and protein (right) represented as log_2_ fold change relative to time zero. All graphs use the same axis units. Data represent averages of results from at least three replicates. Error bars show standard errors of the means. For points with no visible error bars, the bars are within the size of the symbol. (B) NADH dehydrogenase (closed symbols) and lactate dehydrogenase (open symbols). (C) Glucose dehydrogenase. (D) Cytochrome *c* peroxidase. (E) The cytochrome *bd* complex.

Upon exposure to oxygen, Z. mobilis exhibited concerted upregulation of three membrane-bound dehydrogenases. Both transcriptomics and proteomics data show upregulation of a type 2 NADH dehydrogenase (Ndh) (ZMO1113), lactate dehydrogenase (Ldh) (ZMO0256), and glucose dehydrogenase (Gdh) (ZMO0072) (Fig. 9A to C). Together, Ndh, Ldh, and Gdh account for the three dehydrogenase activities that have been previously measured in Z. mobilis membrane fractions (44). Ndh protein levels increased by 4-fold above the anaerobic zero time point after 2 h of oxygen exposure. Given that Ndh competes with alcohol dehydrogenase for NADH as a substrate, increased Ndh expression may be a critical factor leading to the observed deficiency in ethanol production (Fig. 9B). The increase in Gdh protein levels was modest (1.5-fold), indicating that other factors may be involved in the dramatic increase in gluconate production (Fig. 9C).

In addition to membrane-bound dehydrogenases, we also observed upregulation of quinol oxidases upon exposure to oxygen. A cytochrome *c* peroxidase (ZMO1136), whose activity has been shown to improve tolerance to H_2_O_2_ in Z. mobilis, was upregulated with respect to both protein and transcript (Fig. 9D) (18). E. coli cytochrome *c* peroxidase has been shown to serve as a terminal electron acceptor, utilizing electrons from the quinone pool to reduce H_2_O_2_ (45). The only other known terminal oxidase in the Z. mobilis genome is a cytochrome *bd*-type quinol oxidase, comprised of two proteins, CydA and CydB, that are transcribed as an operon (44). Both CydA and CydB were upregulated at the level of transcription, however, whereas protein levels for CydA increased moderately and levels of CydB were highly variable and did not show the typical trend of protein upregulation (Fig. 9E). It is therefore unclear whether upregulation of the *bd*-type terminal oxidase is a physiologically relevant aspect of the response to oxygen. However, given the consistency in the transcript levels, it is possible that the protein signal for CydB was simply difficult to detect above the noise level.

Together, the upregulated dehydrogenases and quinol oxidases comprise all the necessary components to transfer electrons from glucose, lactate, or NADH to hydrogen peroxide or molecular oxygen. The fact that increased gluconate production and decreased ethanol production continue throughout aerobic growth suggests that increased activity of the electron transport chain may be an adaptive response that helps Z. mobilis cope with oxidative stress.

## DISCUSSION

### Iron-sulfur cluster damage critically impacts central carbon metabolism.

Taken together, the data presented in this study indicate that oxidative damage to FeS clusters is a major factor influencing Z. mobilis metabolism and transcriptional regulation during the transfer from anaerobic to aerobic growth conditions. This is consistent with what has been observed in other bacteria. Previous studies have shown that deletion of superoxide-scavenging enzymes in E. coli causes growth defects that can be traced to inactivation of FeS-cluster enzymes, including AcnA and LeuCD (36, 46). Our metabolomics analysis indicates that both AcnA and LeuCD also appear to be temporarily disrupted by oxygen exposure in Z. mobilis (Fig. 6). However, in Z. mobilis, inactivation of the FeS enzymes of the MEP pathway resulted in a much larger metabolic perturbation than did inactivation of AcnA or LeuCD (Fig. 3). It has been proposed that the most immediate source of oxygen toxicity is the poisoning of FeS enzymes (47). The metabolomics data presented in Fig. 3 and 6 support this claim, as do the transcriptomics and proteomics data indicating upregulation of FeS biogenesis and accessory proteins upon exposure to oxygen.

### The importance of auxiliary enzymes in maintaining MEP pathway activity.

The most extreme metabolic change that we observed during the 2-h oxygen exposure time course was in intracellular levels of the MEP pathway intermediate MEcDP. The fact that MEcDP accumulated to levels 45-fold above baseline highlights the extreme sensitivity of the MEP pathway to the redox state and the stability of the FeS-cluster cofactors of IspG and IspH. It also supports the notion that Z. mobilis is capable of sustaining high carbon flux through the MEP pathway. Remarkably, the severe metabolic bottleneck was almost entirely resolved with minimal changes in levels of MEP pathway enzymes following upregulation of flavodoxin reductase and the *suf* operon. These results suggest that such auxiliary enzymes have the capacity to dramatically increase flux through the MEP pathway under conditions where subpopulations of IspG and IspH enzymes are in their oxidized or apo forms. Such is the case during metabolic engineering when IspG, IspH, and other redox-active enzymes of the MEP pathway are artificially upregulated. It is therefore important to consider the auxiliary enzymes that maintain FeS-cluster cofactor activity when performing directed metabolic engineering. In support of our observations, a recent study showed that, in E. coli, overexpression of Fpr and FldA alongside the enzymes of the MEP pathway increased isoprenoid product generation by 4-fold compared to the levels seen with MEP enzymes alone (48). The results of our study indicate not only that this approach is likely to succeed in Z. mobilis but also that upregulation of FldA may not be necessary given that increased activity of the MEP pathway was achieved with no increase in FldA protein levels during the oxygen exposure time course. In fact, maintaining FldA at native levels may improve product generation by limiting the protein burden in engineered cells.

### Upregulation of the electron transport chain as a strategy to mitigate oxidative stress.

The role of the electron transport chain in Z. mobilis remains puzzling as cultures clearly exhibit lower growth rates under aerobic conditions. The results of this study show that enzymes of the electron transport chain are upregulated in response to oxygen, suggesting that at least one role may be to neutralize reactive oxygen species, thereby limiting the toxic effects of oxygen exposure.

The increased expression of membrane-bound dehydrogenase and quinol oxidases was accompanied by a profound metabolic remodeling. Anaerobically, glucose was consumed rapidly via the ED pathway and NADH was consumed primarily by ethanol production (Fig. 8). Aerobically, 18% of glucose consumed was exported as gluconate, likely due in part to increased activity of Gdh. High levels of gluconate secretion may be further stimulated by decreased flux through gluconate kinase (GtnK) during inactivation of Edd, as was observed in Pseudomonas putida during iron limitation (Fig. 7C) (49). However, we did not observe significant changes in expression of GtnK (FDR > 0.02, FC < 1.5).

Upregulation of Ndh together with a decrease in ethanol production indicates that a larger portion of NADH was used as an electron donor for the quinone pool under aerobic conditions. Therefore, it appears that in response to oxygen, electrons are increasingly transferred from glucose, NADH, and possibly lactate (as indicated by the upregulation of Ldh) to the quinone pool, where they can be used by cytochrome *c* peroxidase to reduce H_2_O_2_ and limit the formation of reactive oxygen species (Fig. 9) (45). The *bd*-type terminal oxidase may also play a role in mitigating oxidative stress by reducing molecular oxygen or (potentially) H_2_O_2_, as has been reported in E. coli (50, 51). However, the proteomics data are less conclusive with regard to this branch of the electron transport chain. It should be noted that there is another, incomplete branch of the electron transport chain in Z. mobilis, comprised of a cytochrome *bc_1_* complex and a cytochrome *c* 552 enzyme with no known terminal electron acceptor (18). We did not observe any statistically significant changes in these enzymes at the transcript level (FDR > 0.02, FC < 1.3) and observed only minor changes at the protein level (FC < 1.5).

Our conclusions are supported by previous research on the Z. mobilis electron transport chain which connected activity of dehydrogenases and quinol oxidases to oxidative stress. In particular, deletion of the cytochrome *c* peroxidase in ZM6 resulted in increased sensitivity to H_2_O_2_ and inhibition of aerobic growth (18). Additionally, deletion of Ndh resulted in elevated transcript levels for glutaredoxin 2, superoxide dismutase, and Ldh whereas deletion of catalase resulted in elevated transcription of Ndh (17). Our analysis of metabolism and gene expression following exposure to oxygen therefore supports previous indications that transfer of electrons to the quinone pool contributes to protection from oxidative stress in Z. mobilis.

### Upregulation of uncharacterized genes.

We identified several uncharacterized genes that were upregulated upon exposure to oxygen (see Fig. S4 in the supplemental material). Some of these were positioned in an operon with genes of known function that were also upregulated. ZMO1872 and ZMO1875 are in an operon with glutaredoxin (ZMO1873) and BolA (ZMO1874), and all four genes were upregulated at both the protein and transcript levels (Fig. 4). Interestingly, overexpression of ZMO1875 has also been found to increase growth and ethanol production in the presence of inhibitory lignocellulosic hydrolysate (52). Therefore, it appears either that this operon is involved in a generalized stress response or that lignocellulosic hydrolysate induces oxidative stress in Z. mobilis.

Another uncharacterized gene, ZMO0036, is found in an operon with a “putative phosphotransferase system (PTS) IIA-like nitrogen-regulatory protein” (ZMO0037), located in adjacent to a “sigma 54 modulation protein/ribosome hibernation promoting factor” (ZMO0038). All three of these genes were found to be significantly upregulated. Given that nitrogenase protein levels decrease by over 16-fold during the oxygen exposure time course, upregulation of this operon may be involved in a remodeling of nitrogen metabolism.

Although high-level function of some of these unknown genes can be predicted based on their location in the genome, many are still completely uncharacterized. In particular, ZMO0122, ZMO0286, ZMO0994, ZMO1007, ZMO1563, ZMO1603, and ZMO1750 displayed trends in mRNA and protein levels that strongly suggest that they are involved in the response to oxygen, and yet they have no functional annotation (Fig. S4). More research needs to be done to identify the function of these genes before the effect of oxygen on the overall physiology of Z. mobilis can be fully understood.

### The importance of experimental design.

We examined the acute response to oxygen, conducting multi-omics sampling, as early as 5 min after exposure to oxygen. This was a critical aspect of our experimental design; some of the largest metabolic and transcriptional changes occurred within 15 min and, in many cases, quickly returned to the anaerobic baseline. Although we observed excellent agreement between mRNA and protein levels for upregulated genes with our sampling scheme, if our earliest time point had been 1 h after exposure to oxygen, we would have observed an increase in protein abundance with no change in mRNA levels for many upregulated genes.

The results of this study display the profound influence that environmental factors can have on the activity of metabolic pathways that support bioproduct synthesis. By examining the native response to oxygen exposure in Z. mobilis, we identified key potential targets for metabolic engineering to increase production of isoprenoids and higher alcohols. We found that upregulation of IspG, Fpr, and the *suf* operon coincided with enhanced flux through the MEP pathway. These results can help inform metabolic engineering of Z. mobilis to generate high-grade fuels and commodity molecules.

## MATERIALS AND METHODS

### Culture conditions.

Zymomonas mobilis ZM4 (ATCC 31821) was struck onto rich medium-glucose plates (20 g/liter glucose, 2 g/liter KH_2_PO_4_, 10 g/liter yeast extract, 18 g/liter agar) from a frozen 50% glycerol stock and incubated at 30°C anaerobically for 3 to 6 days. A single colony was used to inoculate liquid rich media (20 g/liter glucose, 2 g/liter KH_2_PO_4_, 10 g/liter yeast extract). The colony was grown in the rich media overnight and then subcultured through two passages in *Zymomonas* minimal media [20 g/liter glucose, 1 g/liter KH_2_PO_4_, 1 g/liter K_2_HPO_4_, 0.5 g/liter NaCl, 1 g/liter (NH_4_)_2_SO_4_, 0.2 g/liter MgSO_4_ · 7 H_2_O, 0.025 g/liter Na_2_MoO_4_ · 2 H_2_0, 0.025 g/liter FeSO_4_ · 7 H_2_O, 0.01 g/liter CaCl_2_ · 2 H_2_O, 1 mg/liter calcium pantothenate]. The second minimal medium overnight culture was then used for inoculation of all experimental cultures with a starting optical density at 600 nm (OD_600_) of 0.04 to 0.07 under anaerobic conditions. All experimental cultures were grown in Zymomonas minimal media with an 80-ml starting culture volume in 500-ml Erlenmeyer flasks. Experimental cultures that were exposed to oxygen were grown in baffled flasks to ensure full aeration. Experimental cultures left anaerobic as controls were stirred with a stir bar at 120 rpm. All medium was kept anaerobic for at least 16 h prior to inoculation. The atmosphere in the anaerobic glove bag was composed of 1% H_2_ and 5% CO_2_, with the remaining percentage composed of N_2_. Oxygen levels were kept below 50 ppm.

### Two-hour oxygen exposure time course.

After inoculation of experimental cultures as described above, cultures were grown until they reached an OD_600_ of 0.3 to 0.45, at which point time point zero extractions were taken. Immediately following time point zero, aerobic (O_2_) cultures were removed from the anaerobic glove bag and placed in a 30°C water bath under atmospheric conditions where they were shaken at 250 rpm for the duration of the 2-h time course. Anaerobic (An) cultures remained in the glove bag. Extractions were taken for metabolomics, proteomics, or transcriptomics analysis at *t* = 0, 1, 5, 10, 15, 30, 45, 60, and 120 min after O_2_ cultures were transferred.

This experimental design was followed four separate times. The iterations differed in both replicate number and exact time point sampling scheme (see Table S3 in the supplemental material); however, each iteration lasted 2 h and included at least 3 O_2_ replicates and 2 An replicates. The first three iterations were performed to collect mRNA samples, proteomics samples, and extracellular metabolite samples, respectively. Intracellular metabolites were extracted alongside all four iterations in order to ensure a reproducible physiological response. Intracellular metabolite values matched well from iteration to iteration, and the log_2_ fold change values were averaged together for all metabolites except HMBDP and IDP/DMADP. The aforementioned metabolites produced low-quality signals for the first three iterations, so a fourth iteration was conducted specifically to better quantify these metabolites using a slightly modified LC-MS method. The fold change values for HMBDP and IDP/DMADP presented in Fig. 3B therefore represent averages of only the data from the replicates of the fourth iteration.

### Twenty-four-hour oxygen exposure time course.

A modified experimental design was also conducted which differed from the experiment described above in duration and in time of transfer to oxygen. For this experiment, O_2_ cultures were transferred to aerobic conditions at an OD_600_ of approximately 0.1, after only 1 h of aerobic growth. Extracellular metabolite samples were then taken every 2 h for 8 h following the transfer. A final sample was taken 24 h after inoculation.

### Cadmium treatment time course.

For cadmium treatment, experimental cultures were inoculated and grown until they reached an OD_600_ of 0.3 to 0.45, as was done for the 2-h oxygen exposure time course. After time point zero, cadmium chloride dissolved in water was added to cultures to reach a final concentration of 0.15 mM or 0.05 mM Cd^2+^. No cadmium was added to control cultures. All samples were kept anaerobic for the duration of the time course. Intracellular metabolite extractions were taken at 5, 15, 30, 60, and 120 min after addition of cadmium and 30, 60, and 120 min after time zero for controls.

### Intracellular metabolite extraction.

At the time of extraction, 4 to 6 ml of liquid culture was extracted using a serological pipette. In order to separate cells from the media, the culture was rapidly filtered through a 0.45-µm-pore-size nylon filter (Millipore catalog no. HNWP04700) using a vacuum flask fitted with a sintered glass funnel. Immediately after the media passed through the filter, cells were plunged into cold extraction solvent, simultaneously quenching metabolism, lysing cells, and dissolving intracellular metabolites. This was done by placing the filter face down in a small (5.5-cm diameter) plastic petri dish containing 1.5 ml extraction solvent (40:40:20 methanol-acetonitrile-water; all high-performance liquid chromatography [HPLC] grade) that was kept on dry ice. The filter was then rinsed in the extraction solvent to dislodge the remaining cell debris and metabolites. The extraction solvent was then centrifuged at 16,000 × *g* for 3 min. The supernatant was stored at −80°C until analysis by LC-MS.

### Intracellular metabolite sample preparation for HPLC-MS.

To prepare intracellular samples for analysis by LC-MS, the extract from experimental samples was combined with a reference sample comprised of intracellular metabolite extract (collected as described above) from E. coli grown in M9 minimal media containing universally labeled [U-^13^C]glucose (Cambridge Isotope Laboratories item number CLM-1396-PK) as the sole carbon source. The same reference sample was included in all samples from a given experiment in order to correct for instrument variation. The value representing the ^12^C signal for each metabolite was first divided by value representing the fully labeled ^13^C signal to normalize for variation from injection to injection before further analysis was carried out.

Each experimental sample was thawed at room temperature and mixed in a 1:1 ratio with the single reference sample. The mixture (300-to-200-µl total volume) was then dried under N_2_ gas. Samples were concentrated 3× by resuspending in one third the dried volume using HPLC-grade water, subjected to vortex mixing for 10 s, and centrifuged at 16,000 × *g* for 3 min to remove any remaining cell debris. Supernatant (50 µl) was then transferred to an HPLC vial for LC-MS analysis.

### Extracellular metabolite extraction and sample preparation for HPLC-MS and NMR analysis.

At the time of extraction, a 1-ml volume of culture was collected and centrifuged at 16,000 × *g* for 3 min. The supernatant was stored at −80°C until analysis by LC-MS or NMR. To prepare samples for LC-MS analysis, samples were diluted 1:10 in HPLC-grade water and centrifuged at 16,000 × *g* for 3 min to remove any remaining cell debris. Supernatant (50 µl) was then transferred to an HPLC vial for LC-MS analysis. To prepare samples for NMR analysis, 240 µl of fully concentrated sample was combined in an NMR tube with 300 μl 100 mM sodium carbonate buffer (pH 7) and 60 µl 20 mM trimethylsilylpropanoic acid (TSP) dissolved in D_2_O.

### Metabolomics LC-MS method.

The general LC-MS method was performed using a Vanquish ultra-high-performance liquid chromatography (UHPLC) system (Thermo Scientific) coupled to a hybrid quadrupole Orbitrap mass spectrometer (Q Exactive; Thermo Scientific). The chromatography was done using a reverse-phase C_18_ column (Acquity UPLC BEH) (1.7-µm particle size, 2.1-by-100-mm column). Solvent A was 97% H_2_O and 3% methanol with 10 mM tributylamine (TBA) and ∼10 mM acetic acid for a pH of 8.2. Solvent B was 100% methanol. The total run time was 25 min. Flow rate was held constant at 0.2 ml/min. The chromatography gradient was as follows: 5% solvent B for 2.5 min, linear increase to 95% B over 14.5 min, maintenance at 95% B for 2.5 min, linear decrease back to 5% B over 0.5 min, maintenance at 5% B for 5 min. For the general method, eluent from the column was analyzed by MS from the start of the run until 19 min, at which time the flow was directed to waste for the remainder of the run. For better analysis of compounds IDP/DMADP and HMBDP, only the eluent from the time period from 10 to 13 min was analyzed by MS to allow for higher injection volumes. Compounds separated by HPLC were ionized by heated electrospray ionization and analyzed in negative ionization mode with a scanning range of 85 to 1,000 *m/z*. For the specialized IDP/DMADP and HMBDP run, a scanning range of 200 to 300 *m/z* was used.

### Extracellular metabolite quantitation by LC-MS.

Absolute concentrations for extracellular metabolites detected by LC-MS were determined using a four-point calibration curve generated from analytical standards of known concentrations. For metabolites with nonlinear signal response curves, an internal ^13^C-labeled standard was used to determine the absolute concentration. These were glucose, for which [U-^13^C]glucose was used as an internal standard; gluconate, for which [U-^13^C]gluconate was used (Cambridge Isotope Laboratories item number CLM-8781-PK); and pyruvate, for which [1-^13^C]pyruvate was used (Cambridge Isotope Laboratories item number CLM-1082-PK). Due to the high concentrations of glucose in the media, samples were diluted 1:100 rather than 1:10 to quantify extracellular glucose.

### Extracellular metabolite quantitation by NMR.

For quantitation of extracellular ethanol and acetate, 1H spectra were collected on a Bruker Biospin (Billerica, MA, USA) Avance 500-MHz spectrometer (Billerica, MA, USA) fitted with a cryogenically cooled 5-mm QCI (1H/31P/13C/15N) gradient probe with inverse geometry (proton coils closest to sample), using the prepackaged Bruker pulse program for water suppression. In order to obtain accurate quantitative data, the prescan delay was set to 30 s to give the sample sufficient time for T1 relaxation. All samples were run in D_2_O with a known concentration of TSP used as an internal standard for absolute quantitation.

### Metabolomics computational analysis.

LC-MS raw files were converted to mzXML format and visualized using MAVEN (53). Peaks were chosen by comparison with retention times obtained using analytical standards. For intracellular metabolites, the value representing the ^12^C parent signal was divided by the value representing the fully labeled ^13^C signal (derived from the spiked-in ^13^C-labeled E. coli metabolite extract) to normalize for signal variation from injection to injection. The ^12^C-to-^13^C ratio was then normalized by OD to account for variation in culture density along the 2-h time course. These values were then divided by the values representing time point zero for their respective replicates for O_2_ and An samples separately. The log_2_ values representing these ratios were then averaged to obtain the data reported here.

### Protein extraction and sample preparation for proteomics.

At the time of extraction, 4 ml of culture was collected and cells were pelleted by centrifugation for 2.5 min at 16,000 × *g*. The supernatant was discarded, and the pellets were flash-frozen in liquid nitrogen and stored at −80°C until further analysis.

To prepare proteomics samples for analysis by LC-MS/MS, pellets were thawed and cells were lysed by suspension in 6 M GnHCl, followed by addition of MeOH to reach a concentration of 90%. Samples were centrifuged at 15,000 × *g* for 5 min. The supernatant was discarded, and the pellets were allowed to dry for ∼5 min. The pellets were resuspended in 200 µl 8 M urea–100 mM Tris (pH 8.0)–10 mM TCEP–40 mM chloroacetamide and then diluted to 2 M urea in 50 mM Tris (pH 8). Trypsin was added at an estimated 50:1 ratio, and samples were incubated overnight at ambient temperature. Each sample was desalted over a poly(styrene-codivinylbenzene) (PS-DVB) solid-phase extraction cartridge and dried. The peptide mass was assayed with the peptide colorimetric assay.

### Proteomics LC-MS/MS method.

For each analysis, 2 µg of peptides was loaded onto a 75-µm-inside-diameter (i.d.) 30-cm-long capillary with an imbedded electrospray emitter and packed in a 1.7-µm-particle-size C_18_ BEH column (stationary phase). The mobile phases used were as follows: phase A, 0.2% formic acid; phase B, 0.2% formic acid–70% acetonitrile. The peptides were eluted with a gradient of acetonitrile increasing from 0% to 53% B over 100 min followed by a 5-min 100% B wash and 10 min of equilibration in 0% B.

The eluting peptides were analyzed with an Orbitrap Fusion Lumos mass spectrometer. Survey scans were performed at *R* = 2,400,000 with wide isolation analysis at *m*/*z* 300 to 1,350. Data-dependent top-speed (1-s) tandem MS (MS/MS) sampling of peptide precursors was enabled with dynamic exclusion set to 20 s on precursors with charge states 2 to 4. MS/MS sampling was performed with 0.7-Da quadrupole isolation, fragmentation by higher-energy collisional dissociation (HCD) with a normalized collisional energy (NCE) value of 30, analysis in the ion trap using the “rapid” scan speed, with a maximum inject time of 18 ms, and the automatic gain control (AGC) target set to 3 × 10_4_.

### Proteomics computational analysis.

Raw files were analyzed using MaxQuant 1.5.8.3 (54). Spectra were searched using the Andromeda search engine against a target decoy database. Label-free quantitation and match between runs were toggled on, MS/MS tolerance was set to 0.4 Da, and the number of measurements for each protein was set to 1. Default values were used for all other analysis parameters. The peptides were grouped into subsumable protein groups and filtered to reach 1% FDR, based on the target decoy approach. Log_2_-transformed label-free quantitation intensities were further processed to obtain log_2_-fold change values relative to time point zero for O_2_ and An samples separately.

### RNA isolation and mRNA enrichment and sequencing.

At the time of extraction, 6 ml of culture was collected from the culture flask and immediately combined with 6 ml of methanol that had been kept on dry ice in a 15-ml centrifuge tube. The mixture was then centrifuged at 5,000 × *g* for 15 min at −10°C. The supernatant was discarded, and pellets were frozen at −80°C until RNA extraction. Total RNA was extracted at 4°C using an RNeasy minikit (Qiagen catalog no. 74106) and submitted to the University of Wisconsin—Madison Biotech Center for further analysis. RNA purity and quality were assessed by the use of NanoDrop and Agilent QC. Samples with *A*_260_/*A*_280_ values below 1.8 were rerun through the RNeasy column to remove contaminants. Samples were then processed to remove rRNA by the use of an Illumina EpiCentre Ribo-Zero magnetic kit for bacteria. The cDNA library was prepared using the TruSeq Stranded Total RNA library preparation kit. Samples were sequenced on an Illumina HiSeq 2500 instrument with 100-bp single reads.

### Transcriptomics computational analysis.

Reads were quality checked using FastQC (http://www.bioinformatics.babraham.ac.uk/projects/fastqc/). According to the FastQC reports, quality scores remained high throughout the reads and the level of adapter content was below 0.5%. Therefore, reads were not trimmed before alignment. Raw reads in fastq format were aligned to the Z. mobilis ZM4 ATCC 31821 genome (GenBank accession no. CP023715) using bowtie-2 via the use of RSEM software (55). Alignment parameters were set to default values. Estimated read counts were obtained for each gene using the RSEM method “rsem-calculate-expression” with the “bowtie2” option. Differential expression analysis was conducted using edgeR (56). Log_2_ fold change values were calculated using edgeR for each gene at each time point compared to time zero. O_2_ samples were all compared to the O_2_ zero time point, whereas An samples were compared to the An zero time point.

### Statistical analysis.

Metabolomics, transcriptomics, and proteomics data were all subjected to a repeated-measures analysis of variance (ANOVA) test for statistical analysis. For proteomics, log_2_-transformed signal intensity values were used for statistical analysis. For metabolomics, signal intensity values (divided by the value representing the ^13^C internal labeled standard and corrected for OD) were used. For transcriptomics, values representing numbers of transcripts per million were used. The repeated-measures ANOVA test was used to identify metabolites and genes whose abundance or expression level changed in response to oxygen. Statistical significance indicates that at least one time point during the oxygen exposure time course had a mean value that was different from the means at the other time points. This allowed us to obtain a single *P* value for each metabolite or gene rather than a unique *P* value for every time point compared to time zero or an anaerobic control. Anaerobic controls were not included in statistical analysis because, due to limiting costs, anaerobic control samples were not collected at every time point and are therefore not statistically comparable. Time was considered as a categorical rather than continuous variable to avoid choosing a specific relationship (e.g., linear or quadratic) of metabolite abundance or gene expression with respect to time. *P* values were corrected for multiple-hypothesis testing using the Benjamini-Hochberg method to calculate the false-discovery rate (FDR) (57).

### Data availability.

The mass spectrometry proteomics data have been deposited to the ProteomeXchange Consortium (http://proteomecentral.proteomexchange.org) via the PRIDE (58) partner repository with the data set identifier PXD011545. Transcriptomics data have been deposited into the Gene Expression Omnibus with the data set identifier GSE125123 (59). Metabolomics data and other supplemental materials are available on GitHub (https://github.com/AmadorNoguezLab/Zymo-Oxygen).
