# Supplementary figures and images for: Systems-Level Analysis of Oxygen Exposure in Zymomonas mobilis: Implications for Isoprenoid Production
Source: mSystems. 2019 Feb 12;4(1):e00284-18. doi: 10.1128/mSystems.00284-18 (PMC6372839; doi:10.1128/mSystems.00284-18)

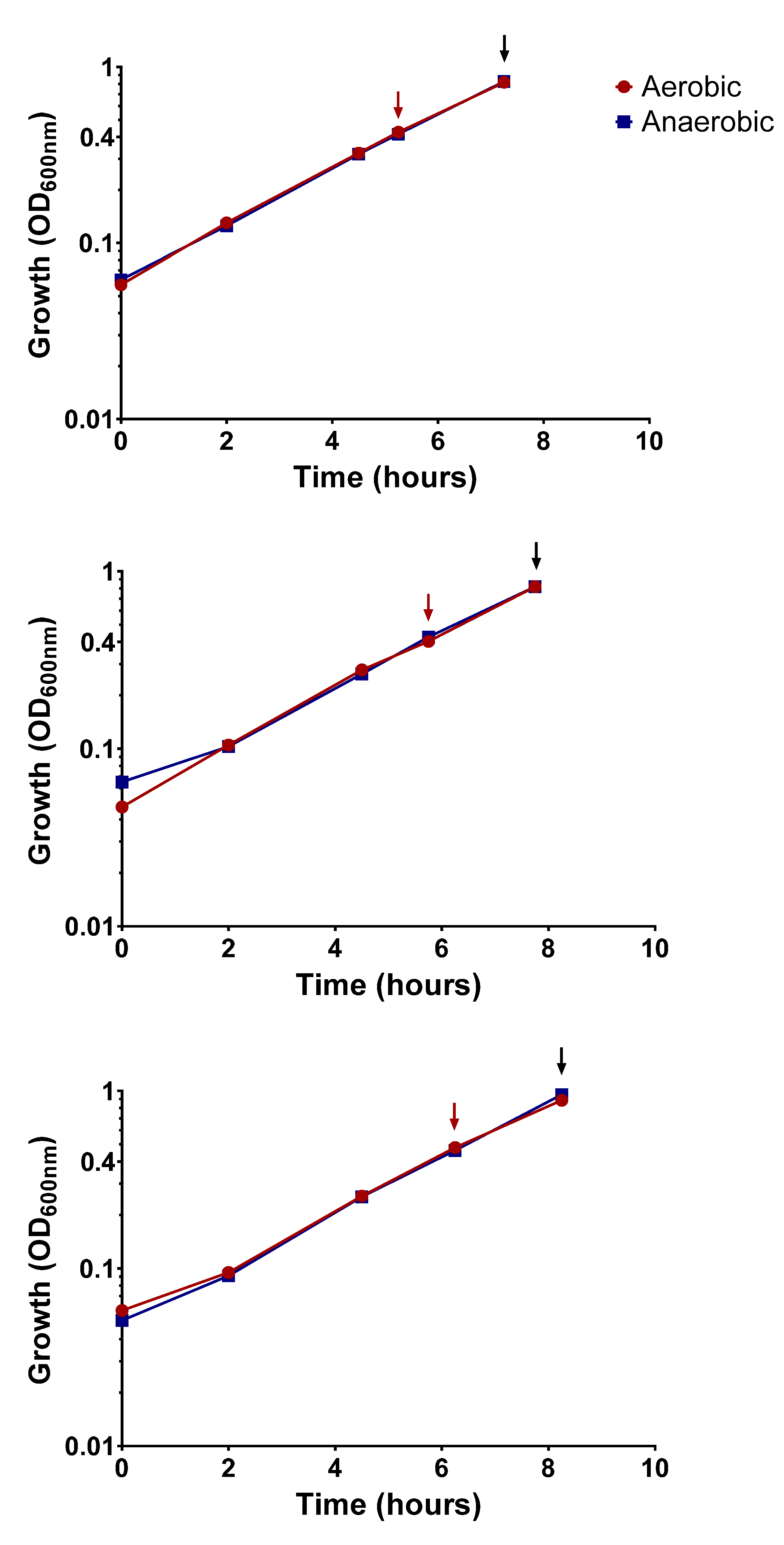

Supplement: FIG S1 [file mSystems.00284-18-sf001.tif]

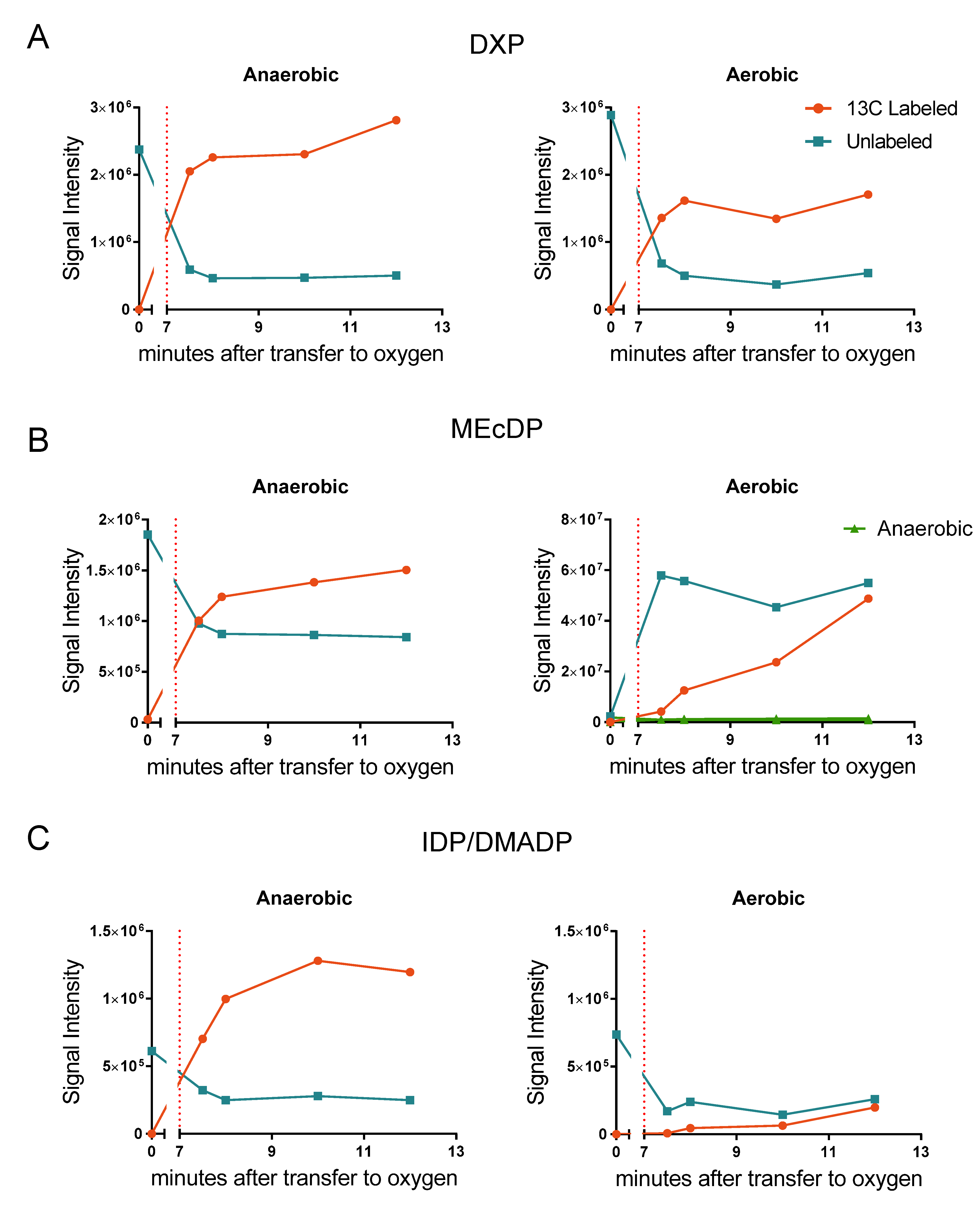

Supplement: FIG S2 [file mSystems.00284-18-sf002.tif]

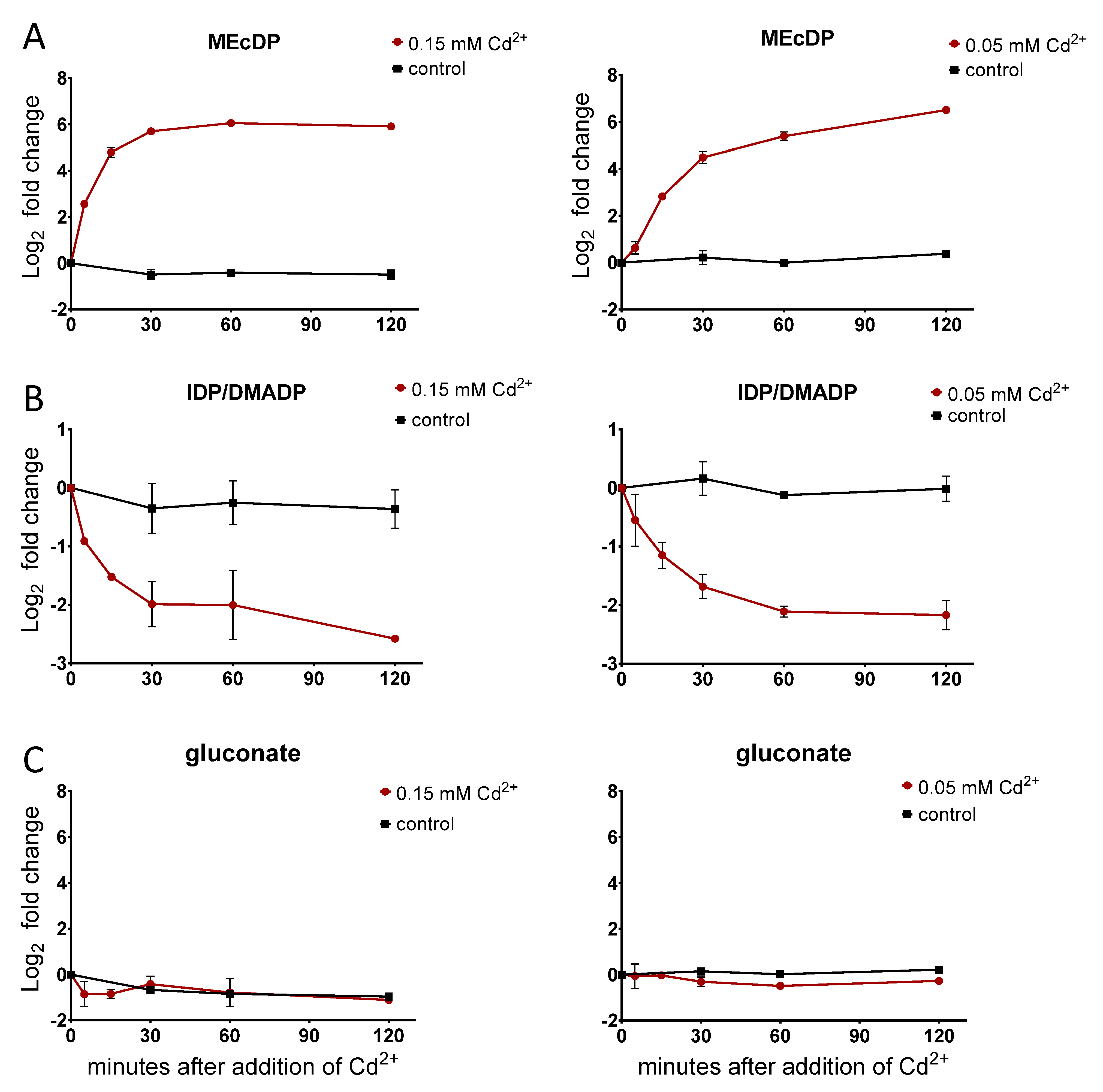

Supplement: FIG S3 [file mSystems.00284-18-sf003.tif]

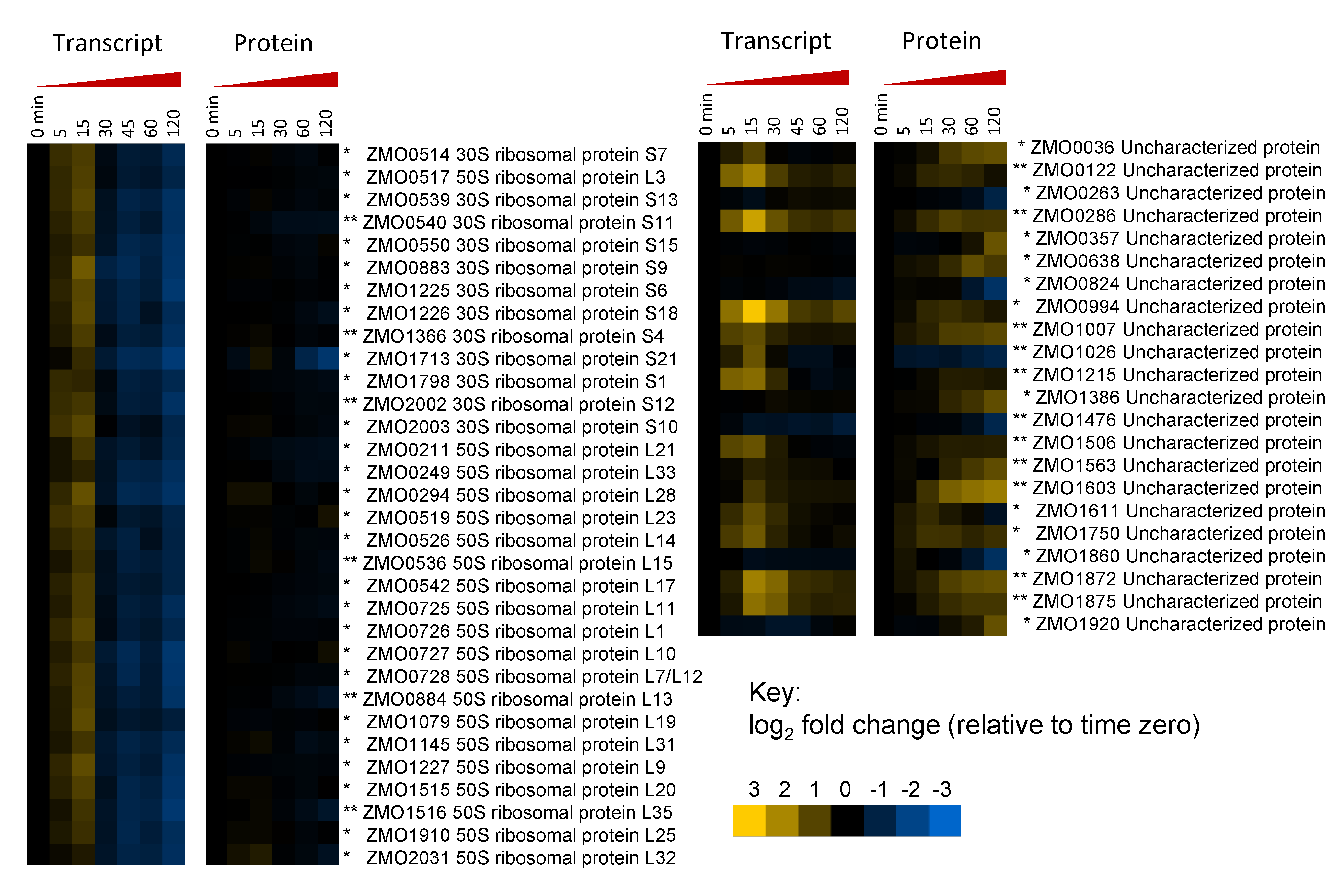

Supplement: FIG S4 [file mSystems.00284-18-sf004.tif]

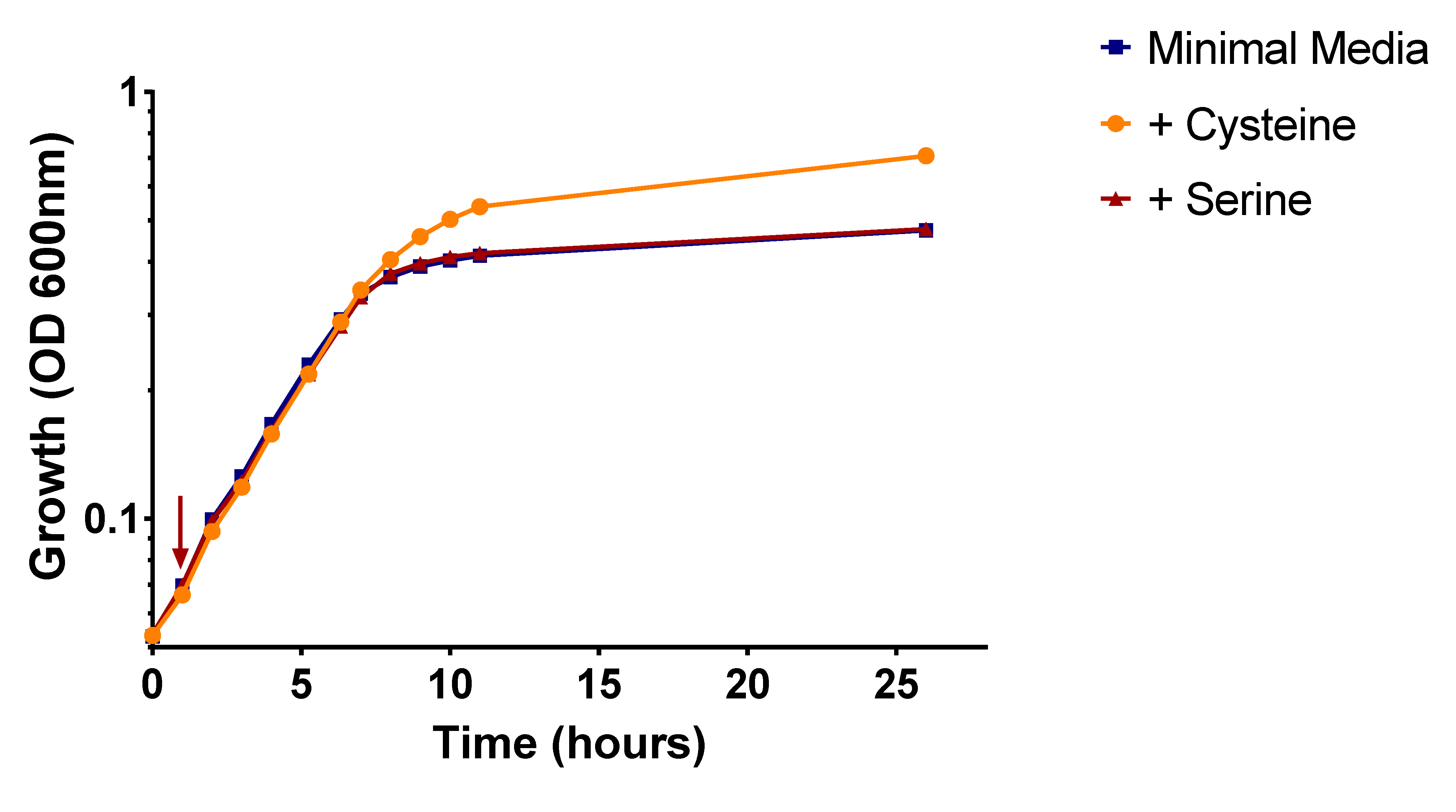

Supplement: FIG S5 [file mSystems.00284-18-sf005.tif]

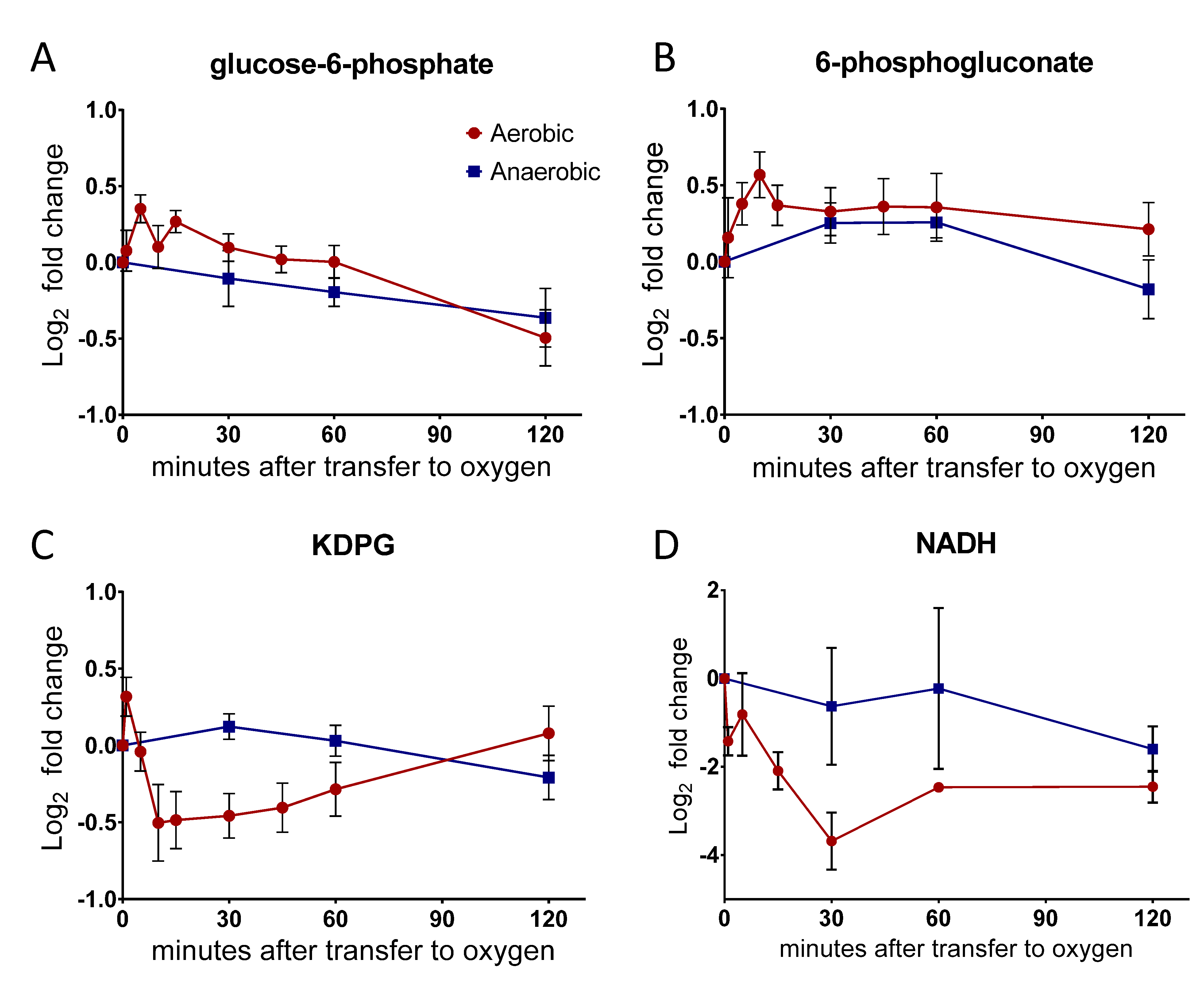

Supplement: FIG S6 [file mSystems.00284-18-sf006.tif]

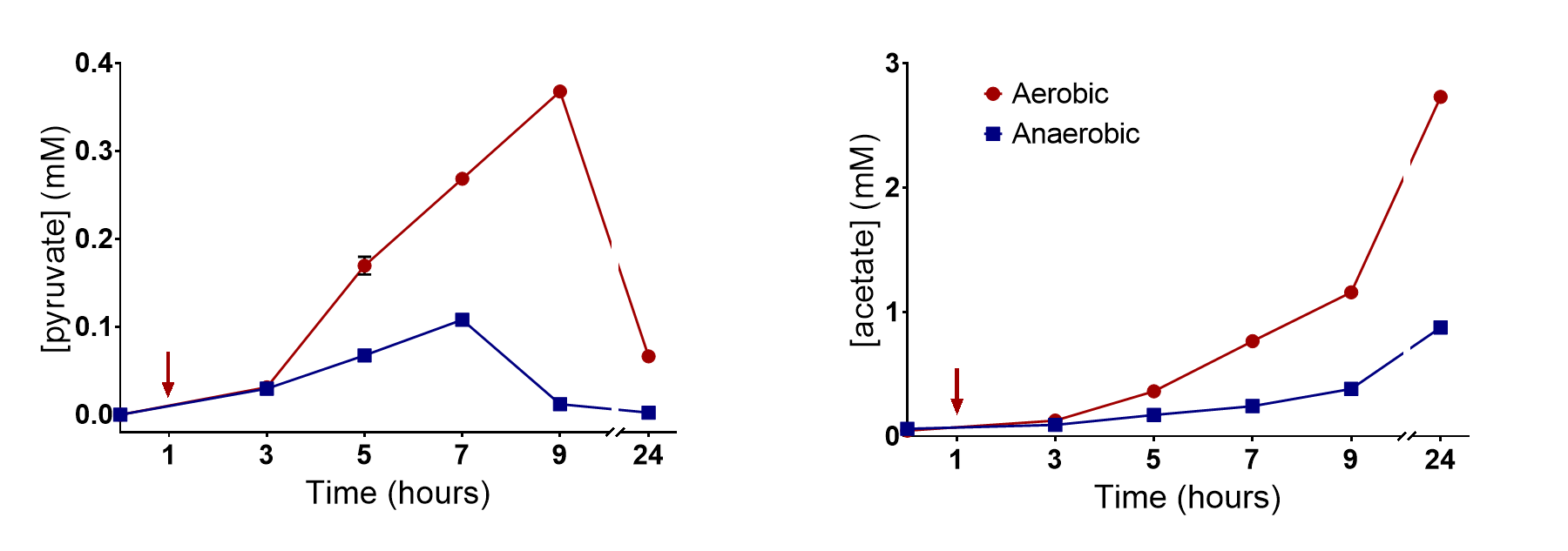

Supplement: FIG S7 [file mSystems.00284-18-sf007.tif]
